# Singlet Fission in Pechmann Dyes: Planar Chromophore Design and Understanding

**DOI:** 10.1021/jacs.4c00288

**Published:** 2024-06-26

**Authors:** Aswathy V. Girija, Weixuan Zeng, William K. Myers, Rachel C. Kilbride, Daniel T. W. Toolan, Cheng Zhong, Felix Plasser, Akshay Rao, Hugo Bronstein

**Affiliations:** † Cavendish Laboratory, University of Cambridge, J.J. Thomson Avenue, Cambridge CB3 0HE, U.K.; ‡ Yusuf Hamied Department of Chemistry, Lensfield Road, Cambridge CB2 1EW, U.K.; § Inorganic Chemistry, University of Oxford, South Parks Road,Oxford OX1 3QR, U.K.; ∥ Department of Chemistry, The University of Sheffield, Sheffield S3 7HF, U.K.; ⊥ College of Chemistry and Molecular Sciences, Wuhan University, Wuhan 430072, PR China; # Department of Chemistry, Loughborough University, Loughborough LE11 3TU, U.K.; ∇ Zhangjiang Laboratory, Shanghai 201210, PR China

## Abstract

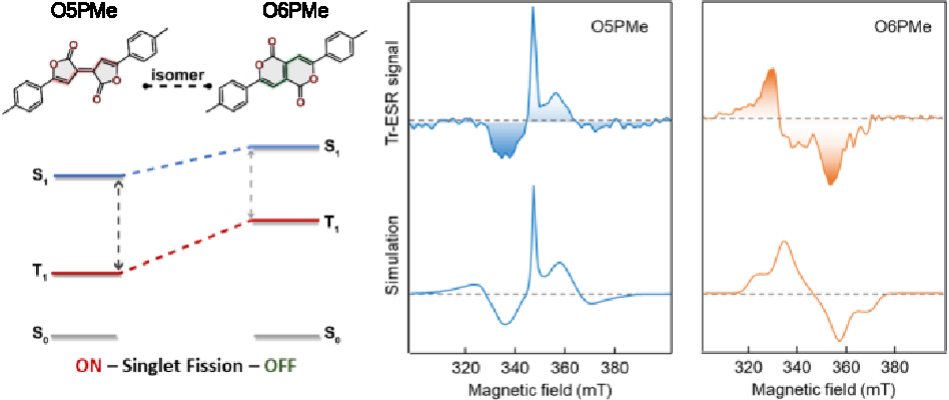

Singlet fission in organic chromophores holds the potential for enhancing photovoltaic efficiencies beyond the single-junction limit. The most basic requirement of a singlet fission material is that it has a large energy gap between its first singlet and triplet excited states. Identifying such compounds is not simple and has been accomplished either through computational screening or by subtle modifications of previously known fission materials. Here, we propose an approach that leverages ground and excited-state aromaticity combined with double-bond conformation to establish simple qualitative design rules for predicting fundamental optical properties without the need for computational modeling. By investigating two Pechmann dye isomers, we demonstrate that although their planarity and degree of charge transfer are similar, singlet fission is active in the isomer with a *trans*-conformation, while the *cis*-isomer exhibits greater favorability for polaronic processes, experimentally validated using ultrafast and electron spin resonance spectroscopy. Our results offer a new design perspective that provides a rational framework for tailoring optoelectronic systems to specific applications such as singlet fission or triplet–triplet annihilation

## Introduction

Singlet fission is a spin-conserving ultrafast photophysical process in organic systems, whereby a high energy singlet (S_1_) exciton splits into two low-energy triplet (T_1_) excitons through an intermediate spin-entangled triplet pair (TT) state. When integrated into photovoltaics, singlet fission offers the opportunity to overcome thermalization losses and thus possesses the potential to substantially improve the efficiency of photovoltaics, surpassing the Shockley–Queisser limit.^1,2^

The key energetic criterion for singlet fission is that the energy of the first excited singlet state must be greater than or equal to twice the energy of the triplet state (*E*(S_1_) ≥ 2*E*(T_1_)).^3^ Additionally, the absolute values of the singlet and triplet states must be considered if they are to be of use for photovoltaic applications.^4^ This has prompted many attempts to develop design rules for tunable materials with large S_1_–T_1_ energy gaps. A closely related application with similar design criteria is triplet–triplet annihilation upconversion (TTA-UC), which requires the S_1_–T_1_ energy gap to be as large as possible and *E*(S_1_) < 2E(T_1_).^5^ By far the most commonly used family of chromophores for both fission and upconversion are the linear acenes where, in general, anthracene, tetracene, and pentacene derivatives all have similar S_1_–T_1_ gaps but sequentially narrowing gaps between the energies of the highest occupied molecular orbital (HOMO) and the lowest unoccupied molecular orbital (LUMO), such that anthracene is used for TTA-UC applications and tetracene and pentacene are more suitable fissionable materials.^6−8^ While the acenes are an incredibly successful series of compounds, their general photoinstability and relatively fixed T_1_ energy mean that alternative materials and design rules are actively sought after. In light of this, different molecular design principles have been explored to expand the pool of chromophores with large S_1_–T_1_ gaps. One of the earliest attempts to do this involves the consideration of biradicaloids as singlet fission candidates,^9,10^ which showed promising results in silico. Others including ourselves have suggested a strategy of exploiting excited-state aromaticity to improve photochemical stability and tune energy levels.^11−14^ Computational material screening has also been undertaken to identify new structures for singlet fission with good success but perhaps at the expense of understanding the underlying rationale for chromophore design.^15,16^

The majority of these proposed design rules and screenings necessitate a large element of computational chemistry, rendering them unsuitable for use as a pen-and-paper tool for organic chemists aiming to design entirely new chromophores. Developments driven by synthetic organic chemistry have led to the breakthrough in the performance of optoelectronic materials, which now demonstrate nearly 20% efficiency in organic photovoltaic devices^17−19^ and a revolution in display technology through organic light-emitting devices.^20−23^ The primary design considerations employed to develop new chromophores for such applications are their planarity and degree of charge transfer (often referred to as the “push–pull” character).

Using only this terminology, organic chemists have created a vast library of chemical compounds that have a diverse range of applications.^15,24^ However, for singlet fission or TTA-UC chromophore design, these considerations are not particularly helpful as it is well-known that the molecules with a large HOMO–LUMO overlap (usually accompanied by high planarity and low charge-transfer character) have the largest S_1_–T_1_ gap and no further possibility for discriminating among these molecules is available.^12,25−27^ As a result, in the regime of planar, non-charge-transfer materials, there is a lack of language and understanding regarding how the molecule’s structure affects its fundamental optical properties such as the S_1_–T_1_ gap and the overall optical gap. While computational screening and design are highly valuable and useful tools, we believe that there is a lack of simple intuitive design rules aimed at developing novel molecules with large S_1_–T_1_ energy gaps. Here, we demonstrate that by combining the use of ground-state and excited-state aromaticity with double-bond conformation, we can describe and qualitatively predict the optical properties of two isomeric organic chromophores known as Pechmann dyes, thereby establishing a new framework for designing materials for singlet fission or other photophysical processes. Additionally, we show that our novel design considerations can be successfully implemented experimentally for the development of singlet fission chromophores.

## Chromophore Design

**Figure 1:**
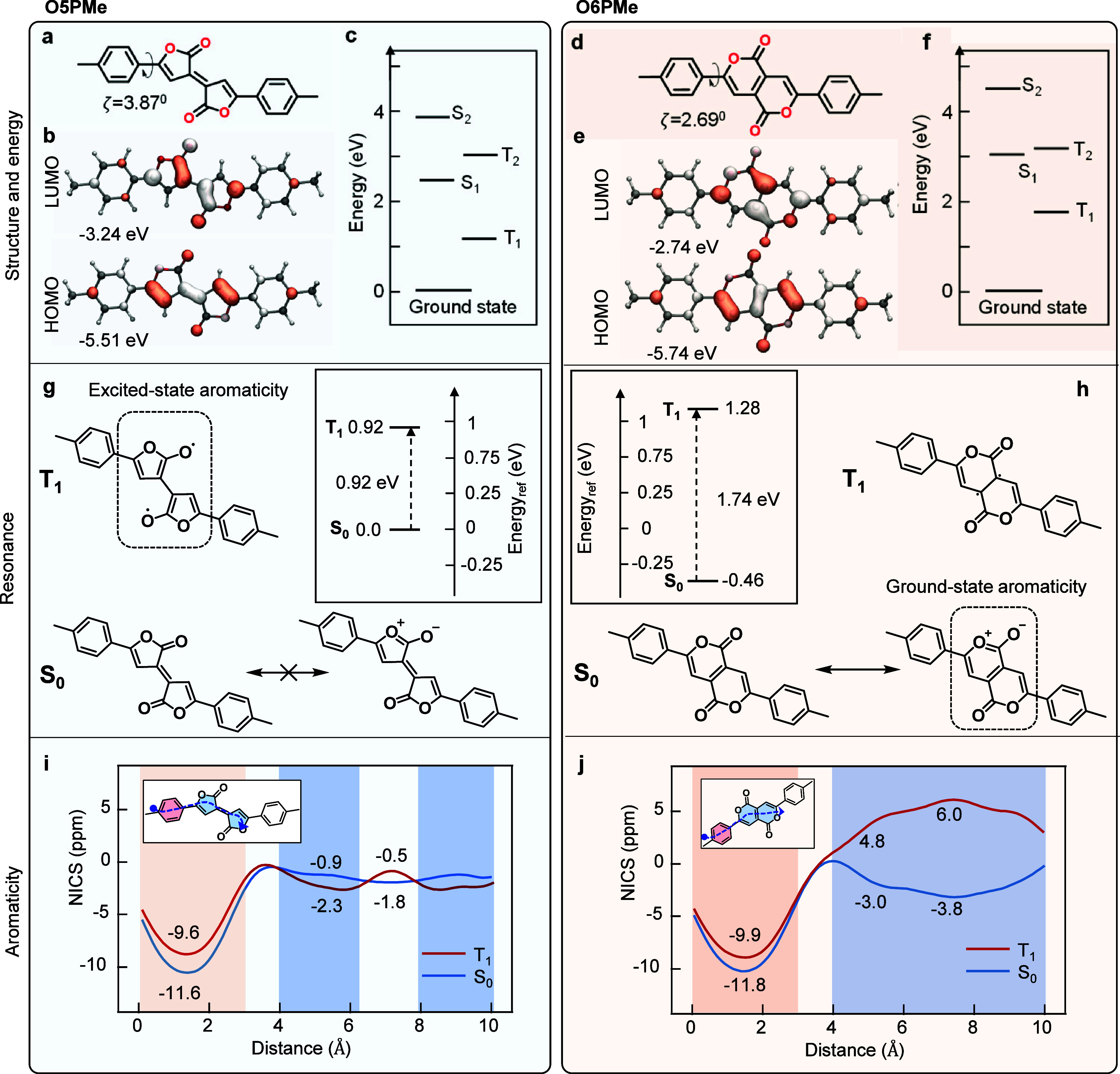
Molecular structures of Pechmann dye isomers (a) O5PMe (left) and (d) O6PMe (right). (b, e) HOMO/LUMO distributions demonstrating the energy gap differences of the isomers and (c, f) calculated energy levels with optimized S_0_ geometry at the (TD)DFT//M06-2X/def2-SVP level. (g, h) Two possible ground-state resonances of the core lactone rings and excited-state biradical structures along with associated relative energies of T_1_, with the S_0_ state of O5PMe as the reference with optimized S_0_ and T_1_ geometries at the DFT//(R/U)M06-2X/def2-SVP level. (i, j) π-NICS-XY scans demonstrating the reversing trends of aromaticity in isomers while going from the S_0_ to T_1_ state with optimized S_0_ geometries at the DFT//(R/U)B3LYP/6-311+G** level.

Pechmann dyes belong to a family of organic chromophores with cross-conjugated lactone rings. The basic structure consists of a 3-butenolide dimer centered about an alkene bridge with benzene rings at the 5 and 5′ positions of the lactone rings (*E*-5,5′-diphenylbifuranylidenedione). Despite being first synthesized in the 1880s as an accidental product,^28−31^ these materials have been underexplored due to their poor solubility. However, their interest for use in the field of organic chemistry has accelerated in the past few years owing to their fluorescence in visible or near-infrared regime,^32^ electron-accepting character,^33,34^ and structural tuneability with various lactone isomers,^35,36^ resulting in interesting optoelectronic features relevant for organic electronics and photovoltaics.^37−43^ Here, we consider the five-membered ring (O5PMe) and six-membered ring (O6PMe) derivatives of Pechmann dyes, as shown in Figure 1a,d, respectively. In both cases, the chromophore is entirely planar and there is no real difference in the degree of charge transfer in the system with the same number of lactone groups. Additionally, both materials are equally conjugated and are highly planar structures with similar sizes. Consideration of the frontier molecular orbitals (FMOs) is also not particularly helpful in differentiating them. However, when looking at their calculated (all ground-state geometries were optimized at the DFT//B3LYP-D3BJ/def2-SVP level, and the excited-state features were obtained with the TDDFT//M06-2X/def2-SVP level) energies of S_1_, S_1_–T_1_, and HOMO–LUMO, we find a striking difference. In the case of the O5PMe, we find that the material has a substantially narrower optical gap (2.67 eV) than O6PMe (3.10 eV). We also find that O5PMe has a larger S_1_–T_1_ gap of O5PMe (1.62 eV) than O6PMe (1.23 eV). Consequently, we identify the former 5-membered ring derivative as a promising singlet fission candidate while the latter 6-membered derivative is better suited for upconversion applications. Interestingly, two material screenings have identified the 5-membered ring form of Pechmann dyes as a singlet fission candidate but with no rationale for its particular electronic structure.^15,16^ Below, we will use these two compounds to try and develop simple design rules that can be used to deduce this information prior to any calculations with the aim of providing both structure–property information for these materials and, more importantly, to provide a new tool for organic chemists to understand and design new materials.

To explain the difference in the optical energy gap *E*(S_1_–S_0_) and the energy gap between the FMOs, we consider the ground- and excited-state aromaticity of the isomers. Two possible ground-state resonances of the lactone rings are shown in the bottom panel of Figure 1g,h, highlighting the possibility of oxygen lone pairs to delocalize into the ring, creating a zwitterionic resonance structure. In the case of O5PMe, the zwitterionic structure would create an unfavorable nonaromatic fulvalene-like structure. Therefore, it can be expected that this resonance structure does not contribute to the ground-state electronic structure. Conversely, in the case of O6PMe, the delocalization of the oxygen lone pairs results in an aromatic 4*n* + 2 electron system. One can imagine this happening on either side of the molecule or both simultaneously (analogous to naphthalene). This suggests a contribution of the right-hand resonance structure as this is an energetically stabilizing resonance structure. The difference can be clearly visualized using XY-scans of the nucleus-independent chemical shifts calculated at 1.7 Å above the ring centers with π-only treatments (NICS(1.7)_π-ZZ_) in Figure 1i,j, which indicate an almost vanishing ring current on the central 5-membered rings (−0.9 ppm) consistent with nonaromaticity, whereas in O6PMe, the central fused rings gain considerable aromaticity (−3 ppm) in the ground state. Additionally, we can deduce that as the ground state in O6PMe is stabilized by aromaticity, its HOMO will be deeper than that in O5PMe. Looking at the excited states, one can assume that the excited state of O6PMe will be antiaromatic in nature because of the reversal of aromaticity criteria as proposed by Baird (4*n* + 2 electrons is aromatic in the ground state but antiaromatic in the excited state) and therefore will have a higher LUMO (and thus a wider optical energy gap), as observed in Figure 1c,f. One can also consider the structure of the resulting biradical formed through simple homolytic fission of the central double bond for both O5PMe and O6PMe, as shown in Figure 1g,h. In the O5PMe isomer, there is formation of a furan heterocycle as opposed to a more quinoidal structure in S_0_, suggesting excited-state Hückel aromaticity, which will act to stabilize the excited states. In contrast, no such aromatic structures can be drawn for O6PMe in the excited-state biradical form. Thus, we can assert that the excited states in O5PMe will be stabilized relative to O6PMe due to the presence of excited-state aromaticity in O5PMe and excited-state antiaromaticity in O6PMe. This is also observed in the NICS-XY scan of the triplet excited state T_1_ in Figure 1i,j, which shows an increase in aromaticity (albeit small) for O5PMe when going from the S_0_ to T_1_ state and a stark change of aromaticity to antiaromaticity in O6PMe when going to the T_1_ state. If one makes a (reasonable) assumption that the configurations of the lowest singlet and triplet excited states are of the same configuration (essentially pure HOMO–LUMO transitions), we can arrive at the conclusion that O5PMe will have a smaller optical energy gap (S_0_–S_1_) than that of the O6PMe. These conclusions based on simple resonance structure considerations are entirely borne out experimentally, showing how powerful this strategy can be.

Now, we turn our attention to the S_1_–T_1_ energy gap in the two isomers. In optoelectronics, materials with both very large and small S_1_–T_1_ gaps are of great interest. Reducing this energy gap is relatively trivial and merely involves the introduction of charge transfer into molecular design. However, in the limit where their HOMO–LUMO overlap is already maximized, it is not straightforward to understand how one can manipulate this value. Time-dependent density functional theory (TD-DFT) predicts that the S_1_–T_1_ gap of O5PMe is larger than that of O6PMe and that the first singlet and triplet states have the same electronic configuration in both compounds (both are predominantly HOMO–LUMO transitions). Additionally, due to their isomeric nature, any differences in the number of electrons or double bonds can be discounted. In the following section, we demonstrate how by looking at the underlying double-bond conformation we can identify the structural origin of this difference in S_1_–T_1_ gap and use it to generate simple design rules that can be applied to predict these gaps without the need for additional calculations.

**Figure 2:**
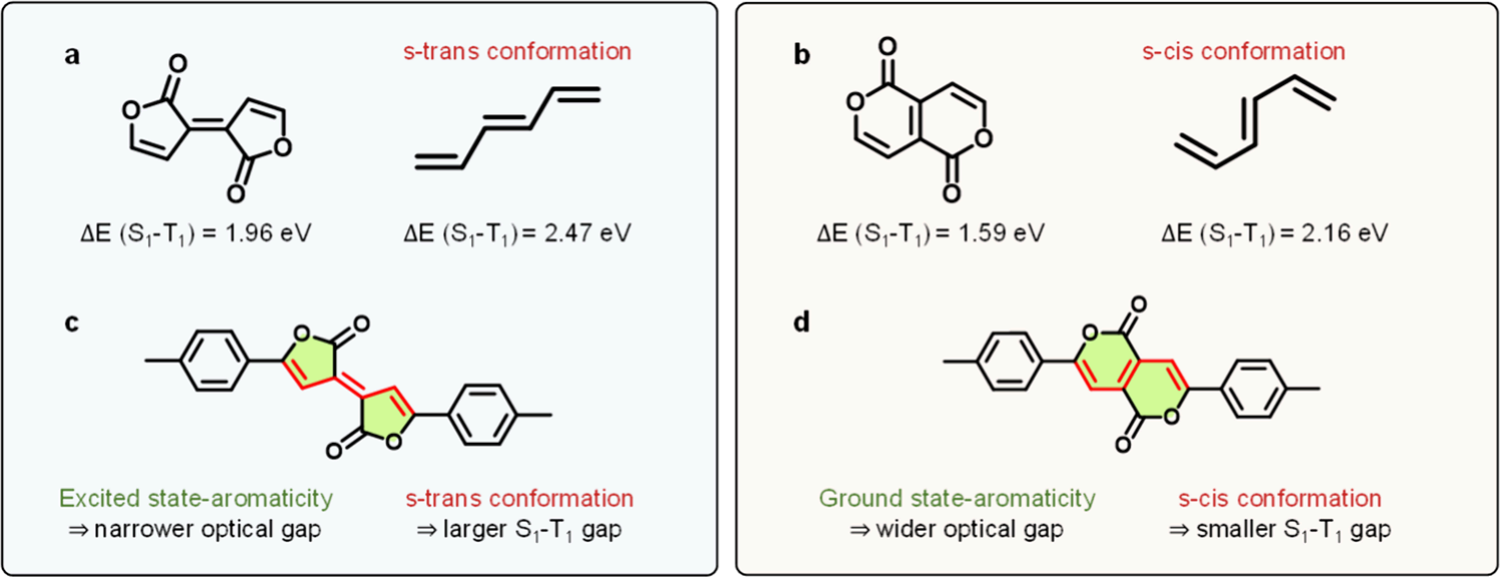
Aromaticity and double-bond conformation of Pechmann dyes. Difference in S1–T1 gap for (a) O5PMe and (b) O6PMe cores and the underlying poly-ene structure. The *trans*-backbone conformation of O5PMe results in a wider S_1_–T_1_ gap compared to O6PMe having a *cis*-conformation. (c, d) Structural representation of aromaticity and its influence on optical energy gaps calculated with optimized S_0_ geometries at the TDDFT//M06-2X/def2-SVP level.

When considering materials with large S_1_–T_1_ energy gaps, it is important to remember that simple linear poly-enes have the largest possible exchange interactions. For this reason, they have also been used as singlet fission materials despite their other drawbacks such as reduced photostability and the presence of dark states. However, when comparing next-generation singlet fission materials to classical poly-enes, one can clearly see that structurally, there are many similarities. Indeed, comparing the HOMOs of O5PMe and O6PMe, one can clearly see that they closely resemble those of diphenylhexatriene (Figure S8). This similarly applies to other chromophoes such as DPP, IND, DPND, and isoindigo, which can all be thought of as-functionalized poly-enes as is shown in Figure S9, and as such, their properties will likely be related somewhat to the parent structure. When comparing the cores of O5PMe and O6PMe, one can easily visualize the underlying hexatriene motif (which is also commonly used in singlet fission derivatives^44,45^). Figure 2a,b shows the difference in the S_1_–T_1_ gap for the two Pechmann dye cores. Remarkably, when we calculate the S_1_–T_1_ gap of the underlying poly-ene structure, we find that these different poly-ene conformers (i.e., rotation about the single bond) have different S_1_–T_1_ energy gaps, as shown adjacently in Figure 2a,b. In the all-*s*-*trans-*conformation, we find a substantially larger S_1_–T_1_ energy gap than in the *s*-*cis-*conformation. It is important to note that in all cases, these structures are fully planar and that it is not the double-bond configuration that is changing. In fact, it is interesting to note that if one isomerizes the central double bond (i.e., changes the configuration), there is almost no effect on the S_1_–T_1_ energy gap. The underlying quantum mechanical reason for this is nontrivial and it is necessary to investigate the properties of singlet and triplet excited states to understand this.^46^ Work to gain a deeper understanding of this and develop a more general set of design rules is currently ongoing and will be reported separately. Additionally, we examine further eight isomers of Pechmann dyes to verify this phenomena robustly (see Supporting Information Table S1). We can use this observation to develop a powerful and straightforward design rule that “chromophores with *trans*-double-bond conformations in their backbone will have larger vertical S_1_–T_1_ gaps than those with *cis-*conformations”. We can thus re-examine the Pechmann dyes within our framework of aromaticity and bond conformation. We see that in the case of O5PMe, we can draw resonance structures for the excited state that are Hückel aromatic, suggesting that it will have a narrower optical energy gap than O6PMe for which we can draw ground-state aromatic resonance structures. We also see that O5PMe has a *trans-*oligoene backbone conformation, suggesting that it will have a wider S_1_–T_1_ gap than O6PMe. Therefore, having carried out this analysis, we can now discuss the structural origin of the differences of optical properties between these two chromophores using simple pen-and-paper arguments. Finally, it also suggests that the O5PMe material is an excellent candidate to develop further as a singlet fission capable material and that the O6PMe material will unlikely undergo singlet fission.

## Results and Discussion

To validate our material design hypothesis, we turned to synthesis and spectroscopy. One important factor that is outside our previous considerations is the role of chromophore coupling in the singlet fission process. It is well-known that the extent of interaction of adjacent chromophores controls the rates of fission, and as such, even with an idealized singlet fission candidate, the solid-state interactions are of critical importance. The O5PMe material was easily synthesized (see Supporting Information Scheme S1) with a copper(I) catalysis ring-close reaction with a carboxylic acid precursor prepared by a one-step Friedel–Crafts reaction. O5PMe can be transformed to O6PMe by heating (200 °C) in a protic solvent, while O5PMe is also stable as its decomposition temperature is above 300 °C. Single-crystal analysis reveals that that these are planar structures extended from the fused ring core to outer phenyl groups. They show a slip-stack pattern of packing arrangement with a π–π distance of 3.1–3.5 Å between the closest planes of the molecules (Figure S14), making them favorable for singlet fission and/or exciton diffusion processes. Additionally, the cyclic voltammetry curves (Figure S5) of both isomers show a quasireversible one-electron reduction process and irreversible one-electron oxidation process, likely due to the electron-withdrawing nature of the lactone groups. From the onsets of the oxidation/reduction curves, O5PMe and O6PMe possess HOMO/LUMO levels of −5.58/–3.71 eV and −5.75/–3.24 eV, respectively. The FMO gap as well as the electrochemically accessed fundamental gap results in the same trend as we saw in Figure 1.^43^

**Figure 3:**
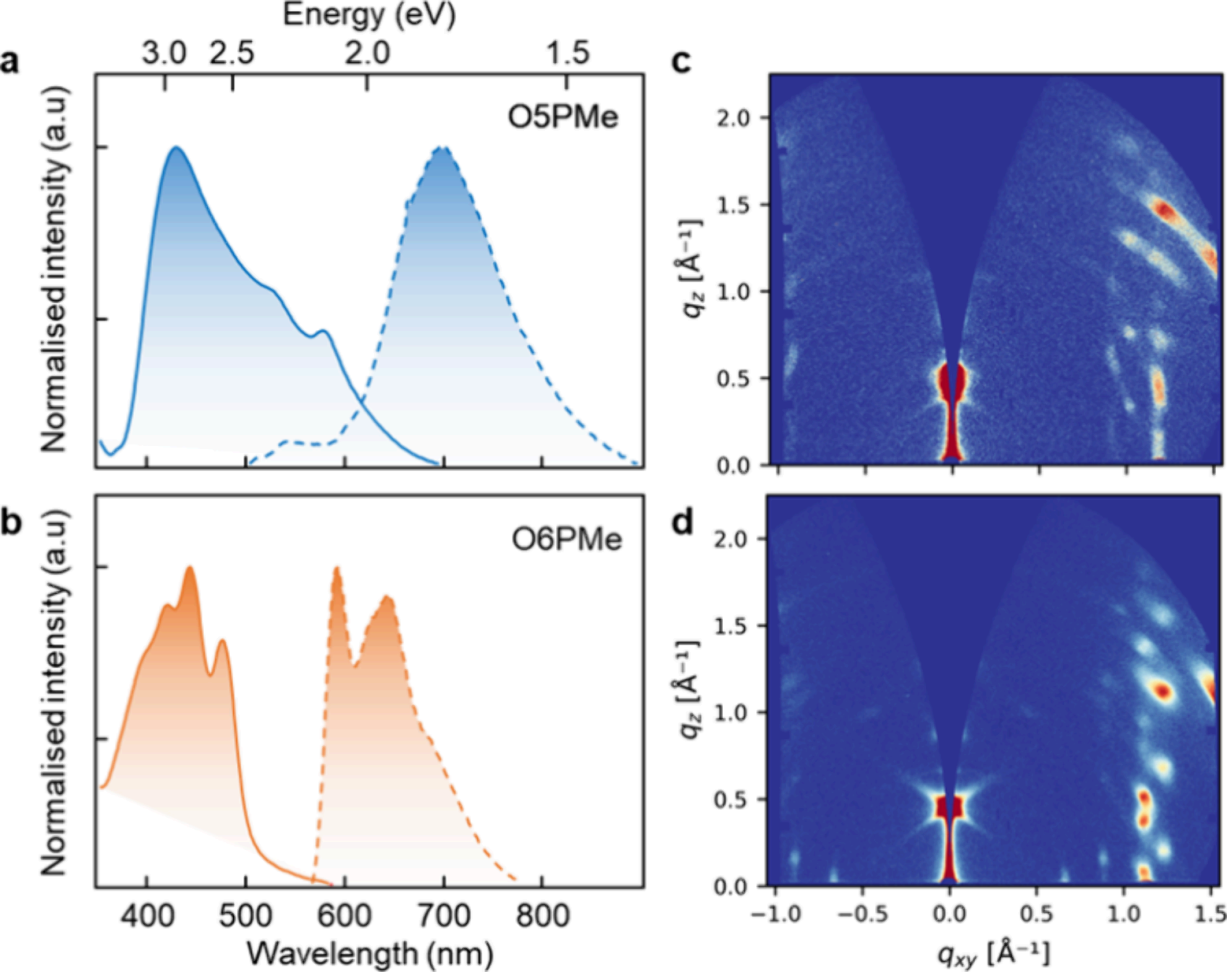
Steady-state linear absorbance (solid lines) and photoluminescence (dashed lines) spectra of 100 nm-thick thermally evaporated films of Pechmann dye isomers (a) O5PMe and (b) O6PMe. 2D GIWAXS patterns of (c) O5PMe and (d) O6PMe films

To study the solid-state characteristics of Pechmann dye isomers, we made thin films by thermal evaporation. Figure 3 shows the steady-state absorbance and photoluminescence spectra of 100 nm-thick O5PMe (Figure 3a) and O6PMe (Figure 3b) films. The absorption spectra of O5PMe, indicated by the blue solid line, are broad and display peaks at 430 nm (2.88 eV), 525 nm (2.36 eV), and 580 nm (2.14 eV), whereas the O6PMe spectra have relatively sharper features with peaks at 420 nm (2.95 eV), 440 nm (2.82 eV), and 475 nm (2.61 eV), which are comparable to the calculated values. The photoluminescence (PL) spectra of O5PMe exhibit broad features having an emission maximum around 700 nm and a suppressed peak around 550 nm, which is not a mirror image of the absorption spectra. The O6PMe emission displays two clearly distinguishable peaks at 600 and 650 nm and is blue-shifted with respect to O5PMe.The broad nature of O5PMe emission beyond 650 nm is suggestive of H-aggregated stacking, improved intermolecular interactions, and the presence of multiple emissive species.^47,48^ We also see that while the absorption spectra of O6PMe in solution state (Figure S14) are comparable to its solid-state spectra, the O5PMe solid-state spectra are hypsochromically shifted with respect to its solution-state spectra, further indicative of H-aggregated stacking in O5PMe films.

The molecular packing and orientation of the films are probed using grazing incidence wide-angle X-ray scattering (GIWAXS) measurements. Both films are highly crystalline and highly oriented with respect to the substrate as evidenced by the appearance of distinct, high intensity Bragg spots in the 2D GIWAXS patterns (Figure 3c,d). The high degree of molecular order observed in the thermally evaporated films make them ideal for validating our material design hypothesis without having to consider contributions from disordered nonequilibrium morphologies. To gain further insights into the thin film molecular packing of O5PMe and O6PMe, the 1D GIWAXS intensity profiles were compared with the simulated powder X-ray diffraction (PXRD) profile from the single crystal structures. The 1D GIWAXS features observed for O5PMe match well with the reflections of the simulated crystal structure, indicating the thin film packing is very similar to the bulk crystal structure (indexing provided in the SI, Figure S15). For O6PMe, however, there are several features present in the 1D GIWAXS profile that do not match with any reflections expected from the simulated PXRD profile of the single crystal structure (see the SI, Figure S16 for further details). The high intensity feature in the out-of-plane (*q*_*z*_ direction) in both 2D GIWAXS patterns corresponds to a length scale of ∼14 Å. This feature has not been observed for previously reported crystal structures; however, it falls outside of the typical *q* range of XRD measurements. Both the intensity and length scale of the *q*_*z*_ scattering feature are likely indicative of the feature being related to the out-of-plane layering of the π–π columnar stacks.

**Figure 4:**
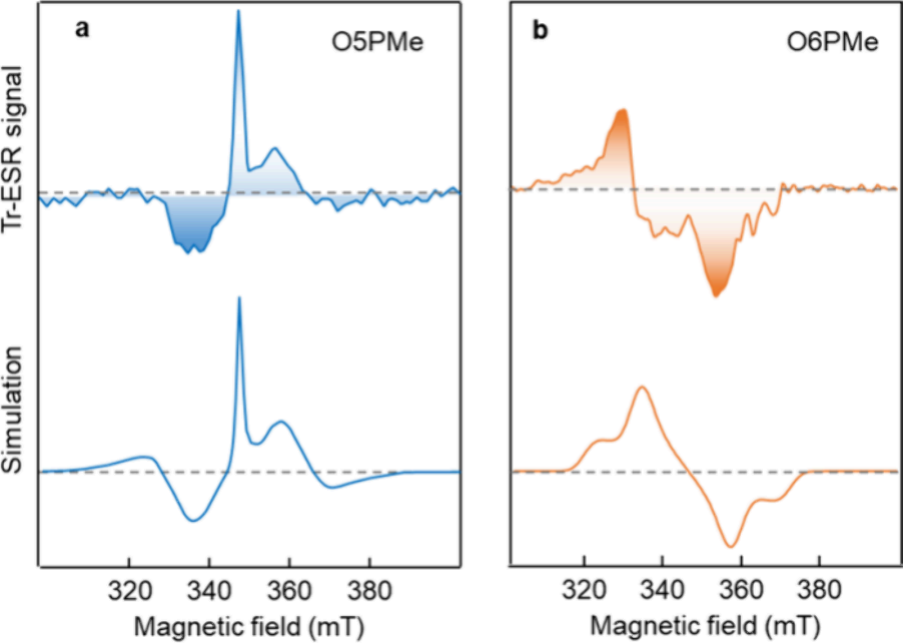
Transient electron spin-resonance spectral features of (a) O5PMe and (b) O6PMe thin films obtained at 80 K (top) and the associated simulated spectra (bottom). Simulation of the O5PMe resulted in a distinct polarization pattern of AEEAAE (A = enhanced absorption, E = emission) indicative of triplets formed via singlet fission. Zero-field splitting (ZFS) parameters: *D* = 780 MHz, |*E*| = 10 MHz, via population of T_0_ eigenstate. The O6PMe simulated spectra shows a AAEE polarization pattern distinctive of polarons with *D* = 735 MHz, |*E*| = 30 MHz and *P_X_*:*P_Y_*:*P_Z_* = 0.4:0.6:0.0.

To investigate the excited-state dynamics in Pechmann dyes, we used time-resolved spin-resonance and optical spectroscopic techniques. Transient electron spin resonance spectroscopy (tr-ESR) is a powerful tool for the detection and characterization of transient photoexcited species. It characterizes the sublevel populations of triplet states and differentiate singlet fission triplets from the triplets formed via intersystem crossing as well as distinguishes free radicals, polaronic species, and charge-transfer states.^49^ The tr-ESR spectra of O5PMe and O6PMe thin films measured at 80 K are shown in Figure 4. O5PMe exhibits a polarization pattern of AEEAAE (A = enhanced absorption, E = emission) evident from the simulated spectra (using EasySpin^50^), which can be attributed to the triplets formed via singlet fission. Simulated zero-field splitting (ZFS) parameters *D* and |*E*| yield 780 and 10 MHz, respectively, with a *g*-value of 2.004 and the T_0_ sublevel preferentially populated at the high field. In the spectrum center, diffuse free charges close to the free-electron *g*-value of 2.0023 are present, which vary strongly with preparation and laser fluence. However, O6PMe thin films exhibit no such signature of triplets arising from singlet fission but instead exhibit a pattern typical of induced polarons or charge transfer states, shown in the bottom panel of Figure 3b, similar to those previously reported P3HT (poly(3-hexylthiophene)) drop-cast films.^51^ Note that the polarization pattern from singlet fission triplets in O5PMe is in line with what has previously been observed for well-known acene-based systems such as pentacene and TIPS-tetracene.^52^ Considering the ZFS parameters and time scales of the process, one can safely eliminate the occurrence of intersystem crossing as a possibility.

**Figure 5:**
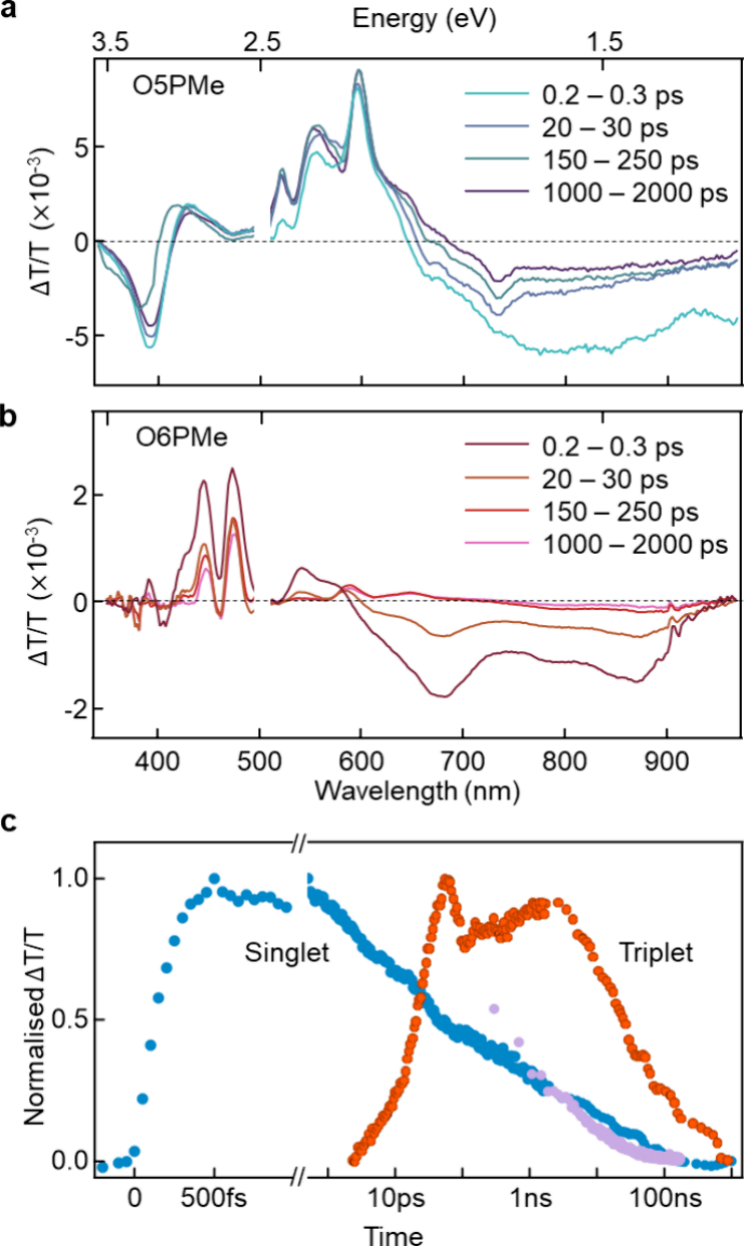
(a) Transient absorption spectra of O5PMe and (b) O6PMe thin films acquired in the time range up to 2 ns in a probe spectral region 340–970 nm when excited with a short 450 nm pump pulse. (c) Spectral kinetics of O5PMe up to 1 μs showing the kinetic profiles of singlet decay (blue) and triplet rise (orange). The singlet decay kinetics matches with the time-resolved photoluminescence decay (purple).

To corroborate the observation of singlet fission occurring in O5PMe but not in O6PMe, as predicted by our design model, we conducted transient-absorption (TA) optical spectroscopy measurements. Figure 5a shows the spectra of an O5PMe film excited by a short pump pulse at 450 nm and probed across a broader range from 340 to 970 nm, within a time window of 200 fs to 2 ns. The changes observed in the differential transmission (Δ*T*/*T*) spectra provide insights into the nature of photoexcited species present in a system, where negative signals correspond to photoinduced absorption (PIA), while positive signals correspond to the ground-state bleach (GSB) as well as stimulated emission (SE) resulting from optical transitions. We observe peaks arising from the ground-state bleach between 400 and 620 nm that match the steady-state absorption peaks, with the latter part also having contributions from stimulated emission.

We also observe a slight redshift of the GSB peaks over time, indicative of relaxation of excited states within the density of states. Additionally, we see a sharp and intense PIA around 390 nm and a broader PIA spectral feature at around 720 nm. These two spectral bands have the same time evolution, implying that they originate from the same photoinduced species. The sharper spectral signatures of this species are consistent with the sharper transitions expected of triplet excitons, and their rapid formation is consistent with the occurrence of singlet fission. Extended time-range measurements reveal that the triplets are long-lived and sustain until 1.1 μs (Figure S20). We further used a spectral deconvolution algorithm to decompose the spectral contributions from different excited-state processes and retrieve two different species (Figure S21) that we attribute to the initially photogenerated singlet state and the subsequently formed free triplets generated via an entangled TT pair state.

In the case of O6PMe thin films, distinct ground-state bleach spectral features are observed between 400 and 500 nm, and spectral evolution points to the formation of a species resembling photoinduced polarons. Subsequent spectral deconvolution analysis yielded not more than one photoexcited species. This would be consistent with the formation and decay of singlet excitons. As previously noted from the tr-ESR spectra that suggested the formation of polarons in this system, we believe that they may contribute to the longer time TA spectra (see Figure S21b). Figure 5c presents the kinetics of the synthesis of O5PMe in two spectral regions 735–745 and 380–395 nm, which have been assigned to singlets and triplets as discussed above. We see that the singlets rise within the instrument response time and decay with a time constant of 20 ps, with a concomitant rise of triplets that continue to evolve beyond 2 ns. We observe that the photoluminescence decay (purple dots, Figure 5c), obtained from time-correlated single photon measurements, matches with the decay of initially photogenerated singlet population.

Taken together, the photophysical measurements clearly show that O5PMe supports singlet fission but that O6PMe does not. This difference cannot be attributed to difference in the packing of neighboring chromophores, instead it is a manifestation of the underlying molecular structure of the two chromophores.

## Conclusions

In this work, we have presented a new perspective for planar organic chromophore design and understanding, employing two Pechmann dye isomers as a model system. By leveraging the concepts of aromaticity and bond conformation, we have shown how basic optical properties such as singlet–triplet energy gap and the optical energy gap, highly relevant in optoelectronics, can be predicted. O5PMe with *s*-*trans-*double-bond conformations in its molecular backbone is found to have a larger S_1_–T_1_ gap compared to its *s*-*cis* counterpart, O6PMe, where the rationale behind this finding can be widely applied to other functionalized poly-ene systems. Furthermore, using steady-state characterization tools and spectroscopy, we gained insights into the crystal packing and orientation in thin films of Pechmann dyes and experimentally demonstrated singlet fission in O5PMe while observing a higher propensity for polaronic processes in the O6PMe isomer. Our results not only pave the path for designing novel and tunable singlet fission systems that can be integrated into photon multiplier systems to enhance photovoltaic efficiencies but also provide valuable guidance for tailoring organic chromophores to suit specific applications.

## Supporting Information Available

Additional calculations demonstrating generality; full synthetic procedures and characterization; and additional time-resolved measurements including crystallographic studies, thin film preparation, grazing incidence wide-angle X-ray scattering, steady state characteristics, time-resolved emission, transient absorption spectroscopy, and transient electron spin resonance (PDF)
