# Supplementary material for: Singlet Fission in Pechmann Dyes: Planar Chromophore Design and Understanding
Source: J Am Chem Soc. 2024 Jun 26;146(27):18253–61. doi: 10.1021/jacs.4c00288 (PMC11240261; doi:10.1021/jacs.4c00288)
Supplement: Supplementary file 1 — ja4c00288_si_001.pdf [file ja4c00288_si_001.pdf]

Singlet Fission in Pechmann Dyes: Planar Chromophore Design and Understanding

Aswathy V. Girija[1], Weixuan Zeng[2,7], William K. Myers[3], Rachel C. Kilbride[4], Daniel T. W. Toolan[4], Cheng Zhong[5], Felix Plasser[6], Akshay Rao[1*] & Hugo Bronstein[2*]

[1] Cavendish Laboratory, University of Cambridge, J.J. Thomson Avenue, Cambridge, CB3 0HE, UK

[2]Yusuf Hamied Department of Chemistry, Lensfield Road, Cambridge, CB2 1EW, UK

[3]Inorganic Chemistry, University of Oxford, South Parks Road, Oxford OX1 3QR, UK

[4]Department of Chemistry, The University of Sheffield, Sheffield S3 7HF, UK

[5]College of Chemistry and Molecular Sciences, Wuhan University, Wuhan, 430072, PR China

[6]Department of Chemistry, Loughborough University, Loughborough, LE11 3TU, UK

[7]Zhangjiang Laboratory, Shanghai, 201210, PR China

Table of Contents

Synthesis 2

General Procedures 2

Elemental Composition 3

Structural Optimization and Excited States 6

Aromaticity 7

Effect of Backbone Conformation on Singlet Triplet Gap 8

Crystallographic Studies 14

Thin-film Preparation 16

Grazing Incidence Wide Angle X-ray Scattering 17

Steady-state Characteristics 19

Time-resolved Emission 20

Transient Absorption Spectroscopy 21

Transient Electron Spin Resonance 23

References 24

Synthesis

General Procedures

^{1}H NMR spectra were recorded on a 400 MHz Avance III HD Spectrometer, 400 MHz Smart Probe Spectrometer or a 500 MHz DCH Cryoprobe Spectrometer using residual protic solvent $CHCl_3$ (δ = 7.26 ppm, s) as the internal standard. ^{1}H NMR chemical shifts are reported to the nearest 0.01 ppm. The coupling constants (J) are measured in Hertz. Mass spectra were recorded using a Waters LCT, Finnigan MAT 900XP or Waters MALDI micro MX spectrometer at the Department of Chemistry, University of Cambridge. Thermal gravimetric analysis was performed under a nitrogen atmosphere at a rate of 10°C min^{-1} using a Mettler Toledo TGA/DSC 2 instrument at a gas flow of 125 cm^3min^{-1}. Reactions that require an inert atmosphere were carried out under argon. Thin layer chromatography (TLC) was carried out on silica gel and visualized using UV light (254, 365 nm). Cyclic voltammetry (CV) was carried out in argon-purged tetrahydrofuran (THF) at room temperature with a PS EmStat4S Pontentiostat analyser. Tetrabutylammonium hexafluorophosphate (TBAPF6) (0.1 m) was used as the supporting electrolyte. The conventional three-electrode configuration consists of a glassy carbon working electrode, a platinum wire auxiliary electrode, and a platinum wire pseudo-reference electrode with ferrocenium–ferrocene (Fc^+/Fc) as the internal standard. Cyclic voltammograms were obtained at scan rate of 100 mV s^{-1}. Formal potentials are calculated as the average of cyclic voltammetric anodic and cathodic peaks. Flash chromatography was carried out on a Biotage® Isolera automated flash chromatography machine on 60 micron silica gel cartridges purchased from Biotage®. FT-IR spectra were recorded on a Perkin Elmer Spectrum One ATR FT-IR spectrometer.

Chemicals

All commercial chemicals were of ≥95% purity and were used as received without further purification. Anhydrous solvents were purchased from Sigma Aldrich or Acros Organics and used as received.

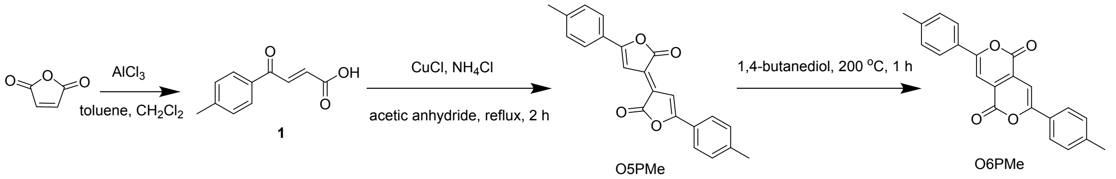

Scheme S1. Synthesis of the Pechmann dyes (**O5PMe** and **O6PMe**).

1 ((*E*)-4-oxo-4-(*p*-tolyl)but-2-enoic acid). To a 100 mL two-necked flask, maleic anhydride (6.13 g, 62.5 mmol) and dry 1,2-dichloroethane (25 mL) were added. After stirring for 10 minutes powdered anhydrous aluminum (16.67 g, 125 mmol) was added and the mixture was stirred for another 20 minutes. Toluene (5.76 g, 62.5 mmol) was then added dropwise and the mixture was stirred for 9 hours at 20 ^oC, 30 minutes at 60 ^oC then refluxed for 30 minutes. The reaction mixture was poured into an ice/water mixture (v/v = 1:1) with 20 mL concentrated

hydrochloric acid. The dichloroethane was removed by steam distillation and then the crude production was collected by filtration. The dissolved crude production by sodium carbonate aqueous solution (at pH 8.5~9.0) filtered off the aluminum hydroxide. The liquor was acidified with hydrochloric acid to pH 1.0. and pure **1** separated out and was collected by filtration to yield a greenish-yellow powder (5.31 g, 27.9 mmol, 46%).

O5PMe. To a 250 mL round-bottomed flask under argon, **1** (1.60 g, 8.41 mmol), CuCl (0.30 g, 3.00 mmol), NH_4Cl (0.32 g, 6.00 mmol) and acetic anhydride (20 mL) were added. The mixture was heated to 140 ^oC and refluxed for 2 hours. After cooling down to room temperature, the crude production was collected by filtration and extracted by refluxed toluene to yield a dark purple powder (779 mg, 2.26 mmol, 54%). R_f = 0.70 (dichloromethane:*n*-hexane v/v = 1:1) ^{1}H NMR (400 MHz, Chloroform-*d*) δ 7.76 (s, 2H), 7.74 (s, 2H), 7.51 (s, 2H), 7.33 (s, 2H), 7.31 (s, 2H), 2.46 (s, 6H). HRMS (ASAP): *m/z* 345.1122 [M-H$^+$]. Calcd. for $C_{22}H_{17}O_4$+ : 345.1127. Structure supported by X-ray crystallography.

O6PMe. To a 150 mL round-bottomed flask under argon, **O5PMe** (345 mg, 1.0 mmol) and 1,4-butanediol (20 mL) were added and heated to 200 ^oC for 1 hour. After cooling down to room temperature, the reaction mixture was poured into saturated aqueous of NaCl and extracted with chloroform, concentrated to yield the crude product. Afterwards, the product was purified by flash column chromatography with aluminum oxide (63-200 micron, dichloromethane) to yield yellow powder (155 mg, 0.45 mmol, 45%). ^{1}H NMR (400 MHz, Chloroform-*d*) δ 7.80 (d, *J* = 1.9 Hz, 2H), 7.79 (d, *J* = 1.9 Hz, 2H), 7.31 (s, 2H), 7.28 (d, *J* = 2.1 Hz, 4H), 2.43 (s, 6H). HRMS (ASAP): *m/z* 345.1117 [M-H$^+$]. Calcd. for $C_{22}H_{17}O_4$+ : 345.1127. Structure supported by X-ray crystallography.

Elemental Composition

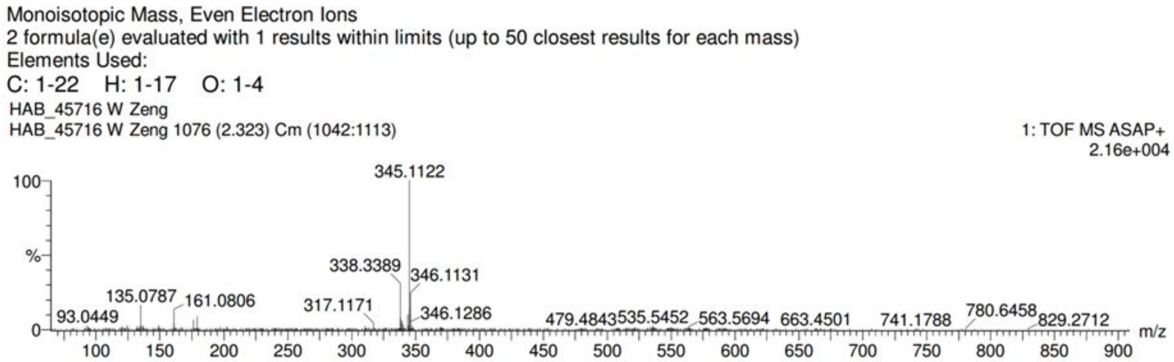

Minimum:				-1.5				
Maximum:		5.0	5.0	50.0				
Mass	Calc. Mass	mDa	PPM	DBE	i-FIT	Norm	Conf(%)	Formula
345.1122	345.1127	-0.5	-1.4	14.5	265.6	n/a	n/a	C22 H17 O4

Figure S1. High resolution mass spectra of **O5PMe**.

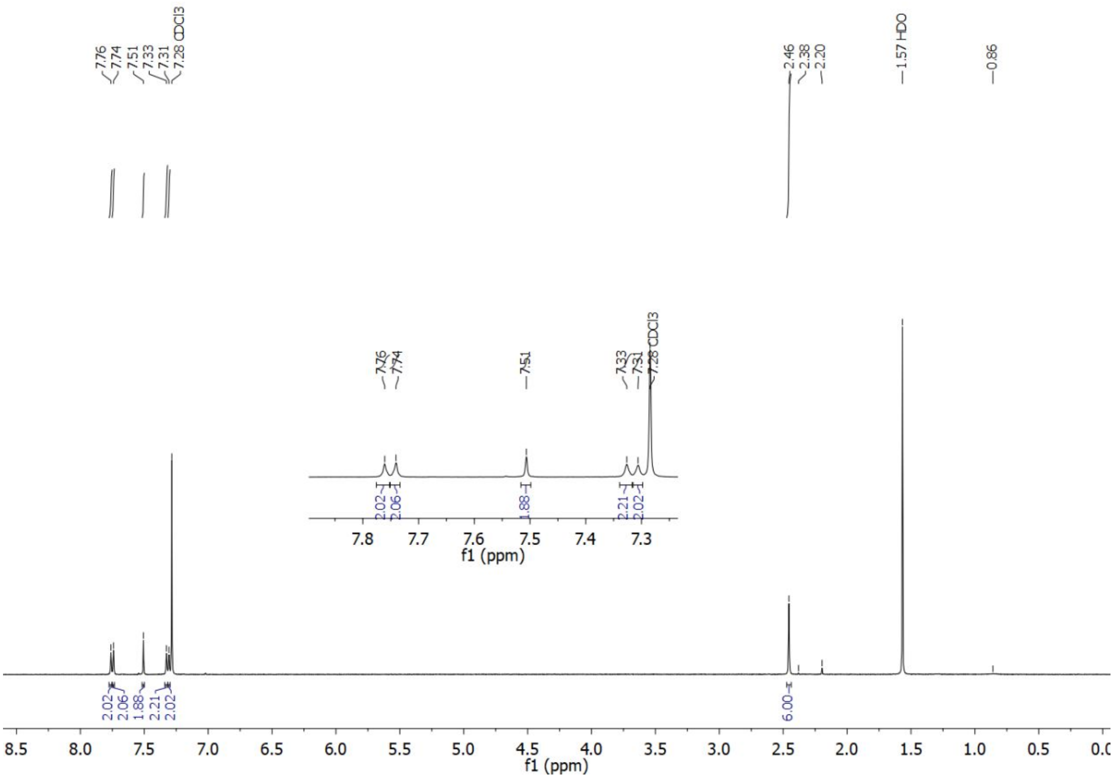

Figure S2. ^{1}H NMR spectra of **O5PMe** (400 MHz, $CDCl_3$, 25 ^oC).

Monoisotopic Mass, Even Electron Ions
2 formula(e) evaluated with 1 results within limits (up to 50 closest results for each mass)
Elements Used:
C: 1-22 H: 1-17 O: 1-4
HAB_45717 W Zeng
HAB_45717 W Zeng 1721 (3.709) Cm (1712:1740)

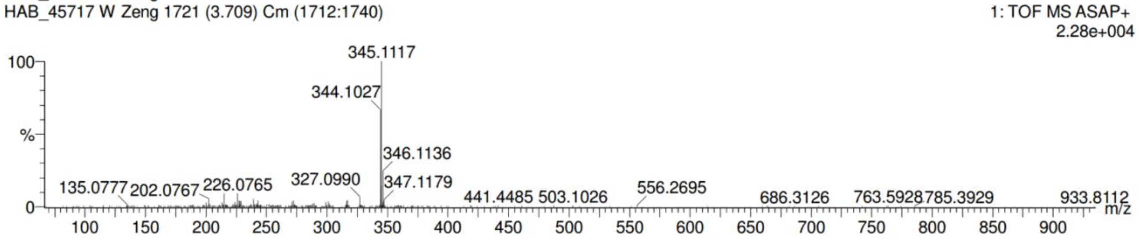

Minimum:				-1.5				
Maximum:		5.0	5.0	50.0				
Mass	Calc. Mass	mDa	PPM	DBE	i-FIT	Norm	Conf(%)	Formula
345.1117	345.1127	-1.0	-2.9	14.5	191.8	n/a	n/a	C22 H17 O4

Figure S3. High resolution mass spectra of **O6PMe**.

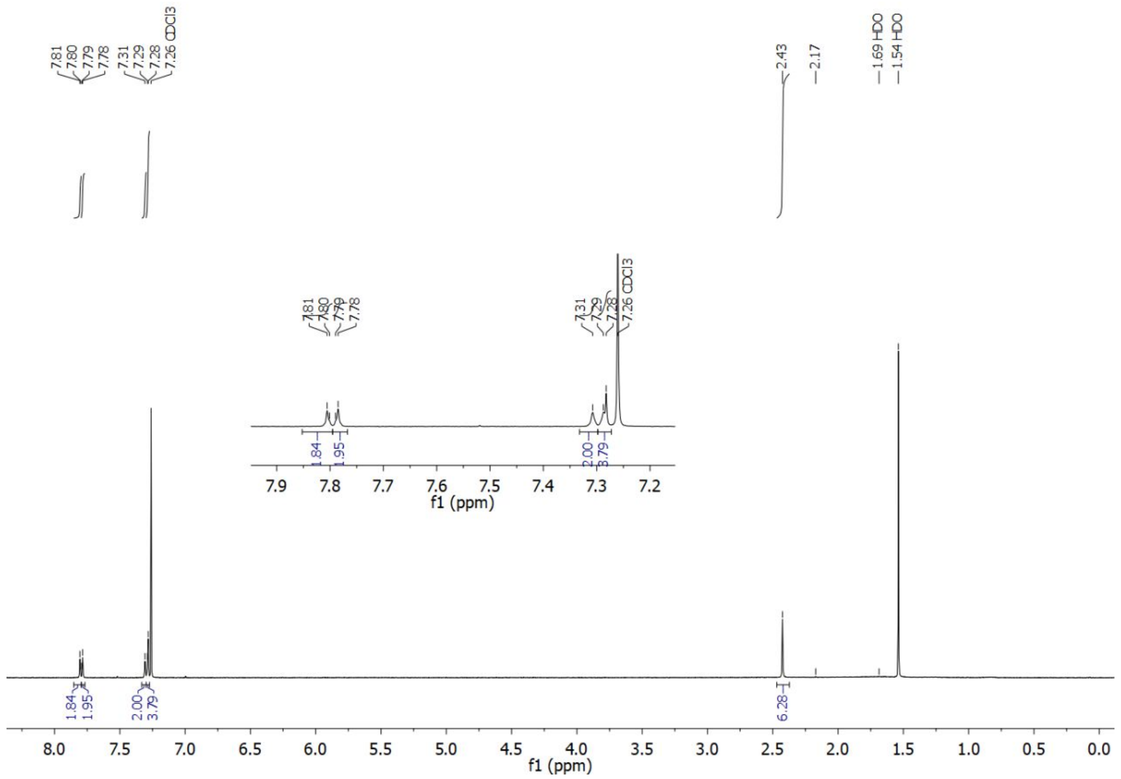

Figure S4. ^{1}H NMR spectra of **O6PMe** (400 MHz, $CDCl_3$, 25 °C).

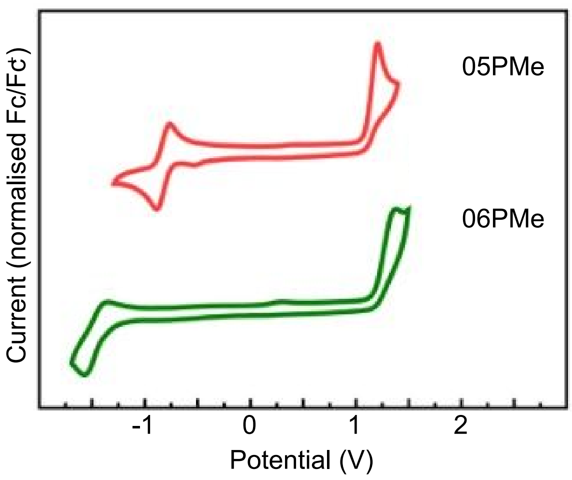

Figure S5. Cyclic voltammograms of **O5PMe** and **O6PMe**.

Structural Optimization and Excited States

The ground state geometries were optimized at DFT//B3LYP-D3BJ/def2-SVP level, and the excited state features were obtained with TDDFT//M06-2X/def2-SVP level. The dispersion correction was conducted using the Grimme's D3 version by the Gaussian 16 program. Excited states analysis was conducted with the TD-DFT results using Multiwfn 3.7 program according to the manual and literature reports.

The 5-member-ring (5-MR) O5PMe exhibits a smaller energy gap between the frontier molecular orbitals (FMOs) with a shallower HOMO and deeper LUMO compared to its 6-member-ring (6-MR) isomer O6PMe. The calculated FMOs show similar distributions at the HOMOs but are quite different at the LUMOs. The energy levels differ with a 0.23 eV gap between the HOMOs and a 0.50 eV gap between the LUMOs. The cyclic voltammetry (CV) curves (Figure S5) of both compounds show a quasi-reversible one-electron reduction process and an irreversible one-electron oxidation process, which can be attributed to their remarkable electron-withdrawing lactone groups.

Based on the onsets of the oxidation/reduction curves, the HOMO/LUMO levels of O5PMe and O6PMe are -5.58/-3.71 eV and -5.75/-3.24 eV, respectively. The FMO gap, as well as the electrochemically accessible fundamental gap, shows the same trend as the optical gap for the compounds, with a smaller vertical excitation energy of 2.67 eV for S1 in O5PMe and 3.14 eV for S1 in O6PMe.[1,2] Due to the significant exchange integral resulting from the large overlap of the FMOs, a large energy difference between E(S_1) and E(T_1) was obtained, resulting in low-lying E(T_1)s for both dyes in various methods.

Aromaticity

Aromaticity was evaluated in terms of the nucleus independent chemical shift (NICS), anisotropy of the induced current density (AICD) plots, the aromatic fluctuation index (FLU), and the multicenter index (MCI) computed at the optimized geometries on B3LYP/6-311+G(d,p) level[3], which had been benchmarked and widely used in evaluating aromatic criteria. NICS values were calculated at 1.7 Å above the ring centres (NICS(1.7)zz) using the gauge independent atomic orbital (GIAO) method. NICS-*XY* scans were performed using the AROMA package scanning from 1.7 Å above the plane of the molecule. AICD plots were generated with the AICD 2.0.0 program at 0.050 a.u. isosurface[4]. The harmonic oscillator model of aromaticity (HOMA) measures the geometric aspect of aromaticity and was calculated with Multiwfn 3.7. The FLU and MCI were performed with the ESI-3D collection of programs[5].

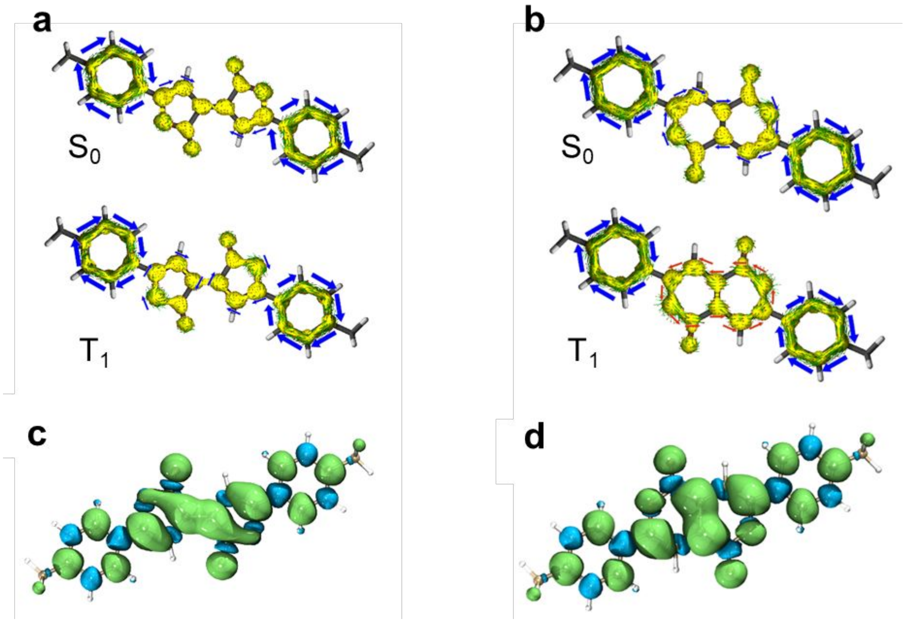

Figure S6: π-Electron ring currents according to AICD in the S_0 and T_1 states of **(a) O5PMe** and **(b) O6PMe** and spin density distribution in the T_1 state of **(c) O5PMe** and **(d) O6PMe**, calculated at (U)B3LYP/6-311+G(d,p) level.

Effect of Backbone Conformation on Singlet Triplet Gap

Diphenylhexatriene

HOMO

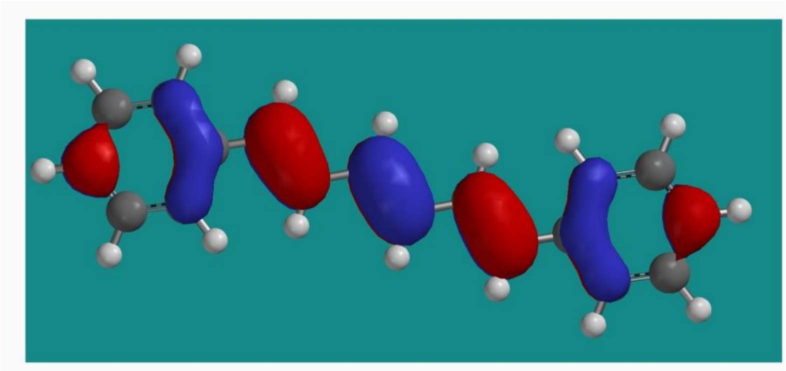

Figure S7: HOMO of diphenylhexatriene

O5PMe HOMO

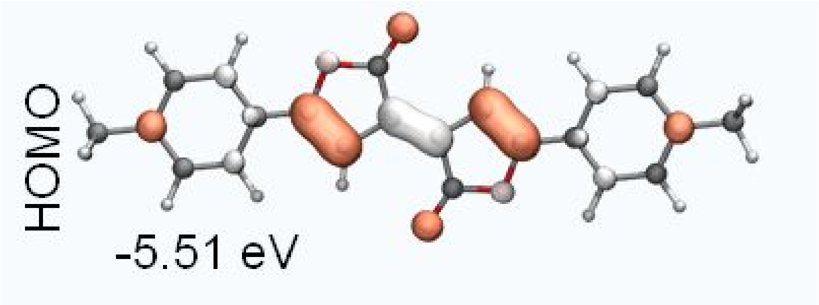

Figure S8: HOMO of O5PMe

The similarity between the HOMO of diphenylhexatriene and the HOMO of both Pechmann dye isomers is clear. Interestingly the HOMOs of the Pechmann dyes appear to be similar to two interacting Phenyl-allyl radicals.

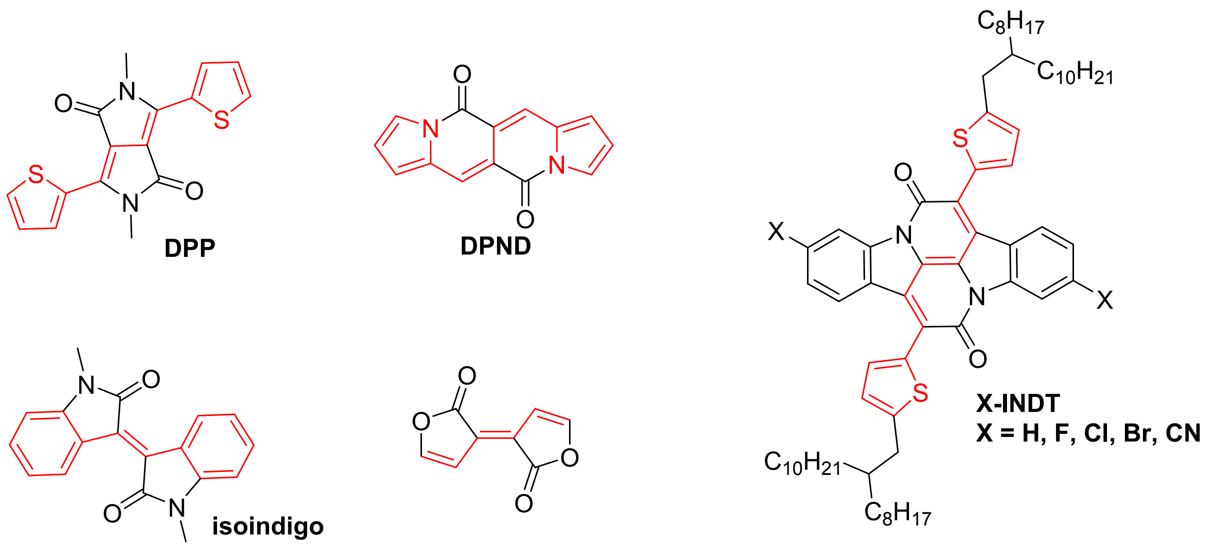

Figure S9: Structural similarities of Pechmann dyes to other material systems such as diketopyrrolopyrrole (DPP), dipyrrolonaphthyridinedione (DPND), diketopyrrolopyrrole thiophene (INDT) and isoindigo, which can all be thought of as functionalised poly-enes as such their properties will likely be related to the parent structure.

Further Analysis on effect of backbone conformation

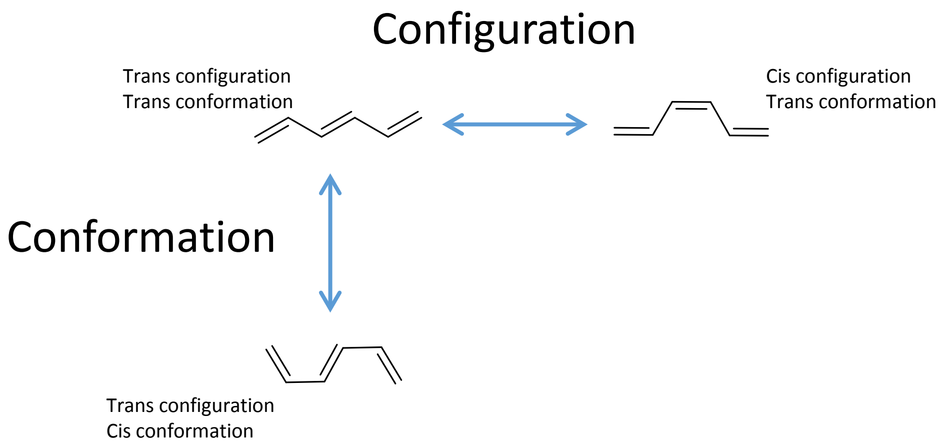

Figure S10: Diagram illustrating the difference between double bond conformation and configuration.

We have now calculated the S_1 and T_1 energy levels of all the sensible isomers of the Pechmann dyes which are the 10 compounds shown below to demonstrate that our proposed design rules are both correct and simple to apply. Alongside each of the Pechmann dye isomers we have also drawn the diphenylhexatriene backbone from which we can apply our rules.

Molecule	structure	T1 (eV)	S1 (eV)	S1-T1 (eV)	Backbone
i55twi	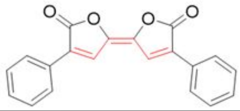	1.351	3.109	1.758	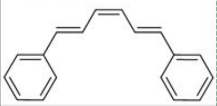
i55	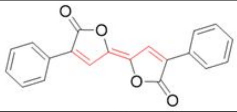	1.3	3.056	1.756	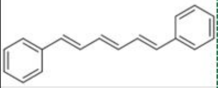
55	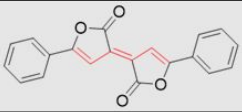	0.854	2.588	1.734	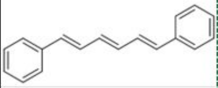
55twi	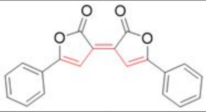	0.96	2.675	1.715	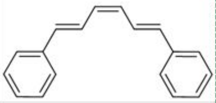
55iso	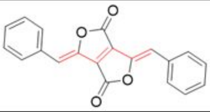	1.512	2.999	1.487	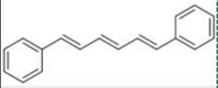
56	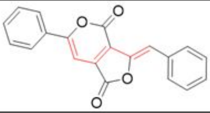	1.66	3.052	1.393	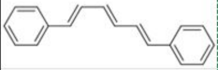
i66	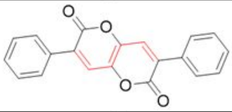	1.752	3.112	1.36	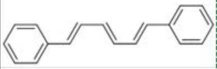
maleic-5	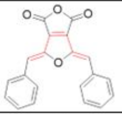	1.143	2.455	1.312	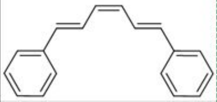
66	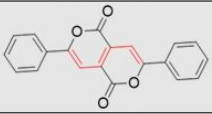	1.789	3.079	1.29	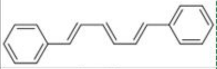
maleic-6	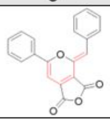	1.277	2.191	0.915	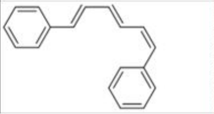

Table S1: Calculated S1-T1 energy gaps of 10 Pechmann Dye isomers and illustration of their underlying polyene backbone conformation/configuration

We can immediately see that as we look at the materials the largest S1-T1 gaps (at the top of the table) these all have trans **conformation** (even though the have differing configurations). As the structures begin to have cis **conformations** in their backbones we can clearly see the S1-T1 gap decrease. We can plot the data to demonstrate this more clearly

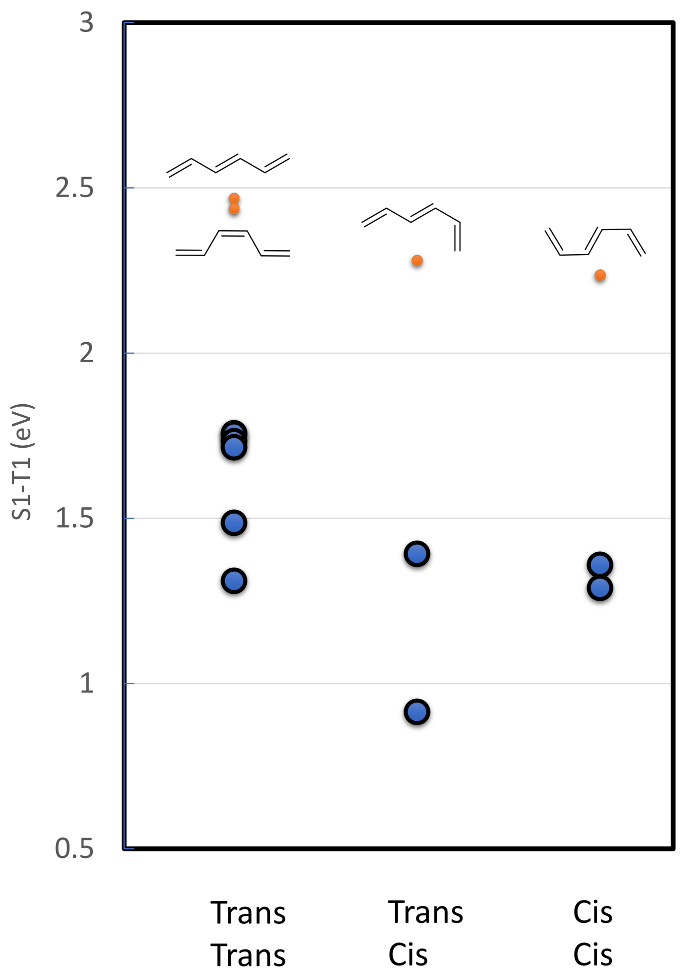

Figure S11: Plot showing decrease in S1-T1 energy gap as structures deviate from all trans backbone conformations

The graph above shows the backbone conformation of the various isomers plotted against their S1-T1 gap (dark blue circles). Above them are the S1-T1 gaps of just the hexatriene motif (yellow circles). It is clear that if you consider the pure hexatriene, as you increase the amount of cis conformer the S1-T1 gap is reduced. It is also clear that this trend is followed very well by the Pechmann dye isomers. We in fact find this rule extremely robust and remarkable considering the structural diversity of the Pechmann dye isomers. By simply drawing the structures and looking at the backbone conformation one would have been able to entirely qualitatively predict which structures has the largest and smallest S1-T1 gaps without the need for any calculations.

Molecule	structure	T1 (eV)	S1 (eV)	S1-T1 (eV)	S1-T1 (eV)	
i55twi	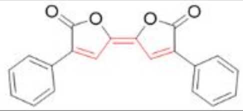	1.351	3.109	1.758	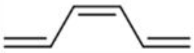	2.536
i55	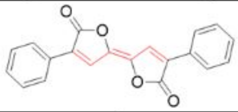	1.3	3.056	1.756	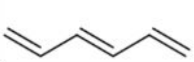	2.567
55	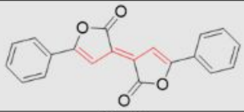	0.854	2.588	1.734	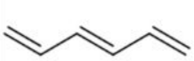	2.575
55twi	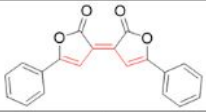	0.96	2.675	1.715	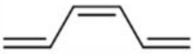	2.567
55iso	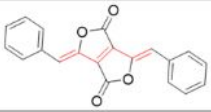	1.512	2.999	1.487	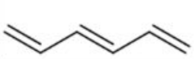	2.519
56	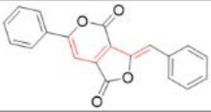	1.66	3.052	1.393	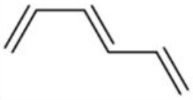	2.298
i66	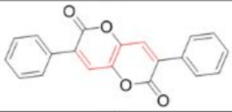	1.752	3.112	1.36	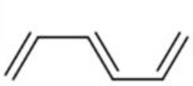	2.235
maleic-5	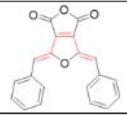	1.143	2.455	1.312	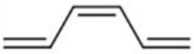	2.307
66	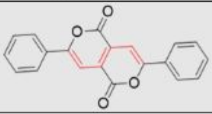	1.789	3.079	1.29	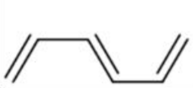	2.227
maleic-6	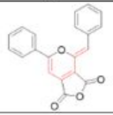	1.277	2.191	0.915	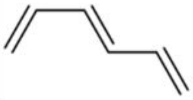	1.949

Table S2: Comparison of S1-T1 gaps of Pechmann dye isomers with those of the underlying hexatriene motif.

We now consider whether the backbone conformation is actually the causation of this. We extracted the exact double bond backbone geometry from the optimised Pechmann Dye isomer, added the correct number of hydrogen atoms and calculated the S1-T1 gaps of these conformers (and configurational isomers).

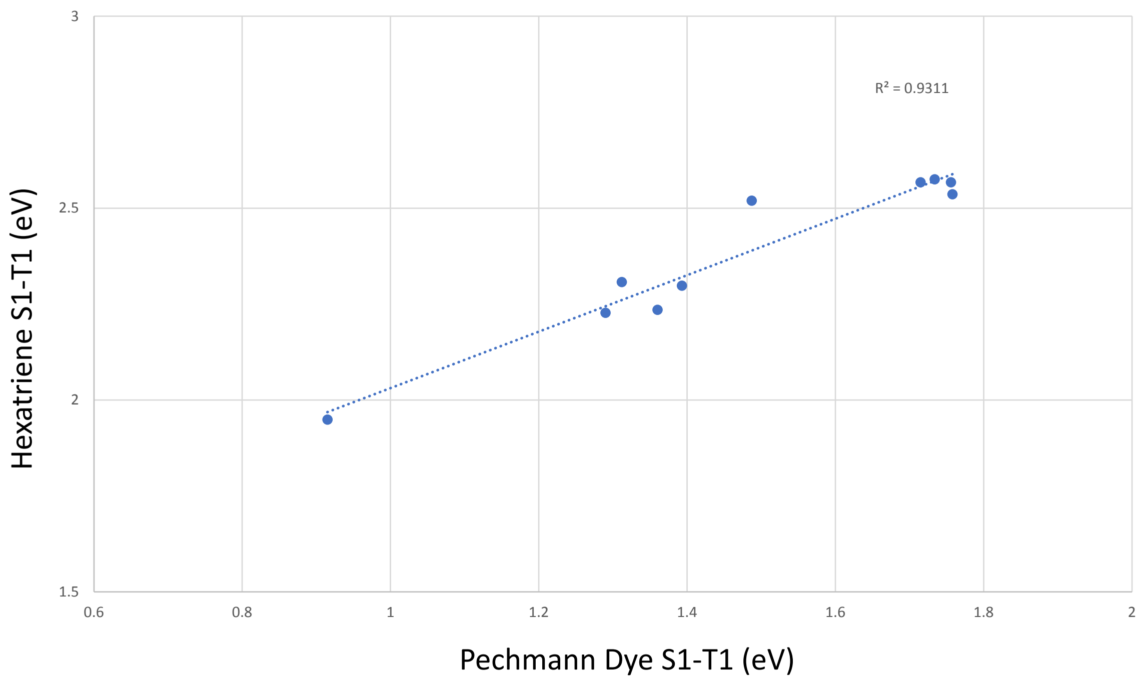

Figure S12: Correlation between S1-T1 gap of Pechmann Dye and Hexatriene S1-T1 energies

When doing this we find an excellent correlation between the two clearly indicating that the size of the S1-T1 gap in the parent hexatriene motif (which is controlled predominantly by its conformation) is directly responsible for the size of the S1-T1 gap in the Pechmann dye. Thus we strongly believe that simply looking at the conformation of these chromophores is indeed a powerful and straightforward method to qualitatively assess the magnitude of the S1-T1 gap.

Crystallographic Studies

X-ray crystallographic data were collected using a D8-QUEST PHOTON-100 diffractometer equipped with an Incoatec IμS Cu microsource (Cu Kα, λ= 1.5418 Å). The temperature was held at 180(2) K using an Oxford Cryosystems N2 cryostat. Data integration and reduction were undertaken with SAINT in the APEX3 software suite. Multi-scan corrections were applied using SADABS.

Table S3. Crystallographic and refinement parameters for **O5PMe** and **O6PMe**.

**Compound**	**O5PMe**	**O6PMe**
chemical formula	C22 H16 O4	C22 H16 O4
M_r / g mol^{-1}	344.35	344.35
crystal system	triclinic	triclinic
space group	$P1^-$	$P1^-$
a / Å	5.7814(4)	6.3031(6)
b / Å	6.7939(5)	7.0806(7)
c / Å	12.0228(8)	9.8009(10)
α / °	94.827(4)	91.269(7)
β / °	103.609(4)	104.925(7)
γ / °	113.541(4)	98.752(7)
V / Å^3	412.28(5)	416.87(7)
Z	1	1
D_c / gcm^{-3}	1.387	1.372
F(000)	180	180
μ(CuK_α) / mm^{-1}	0.777	0.769
T / K	180(2)	180(2)
crystal size / mm	0.28 × 0.20 × 0.01	0.28 × 0.04 × 0.04
index range	−6 → 6<br>−8 → 7<br>−14 → 14	−7 → 7<br>−8 → 8<br>−11 → 11
colected reflections	3961	4170
unique reflections	1447	1478
R_{int}	0.0461	0.0945
reflections with $I > 2\sigma(I)$	1146	891
no. Parameters / restraints	119 / 0	119 / 0
$R(F)$, $F > 2\sigma(F)$	0.0432	0.0605
$wR(F^2)$, $F > 2\sigma(F)$	0.0935	0.1353
$R(F)$, all data	0.0621	0.1064
$wR(F^2)$, all data	0.1021	0.1545
Δ_r (min., max.) e Å^3	-0.255, 0.167	-0.325, 0.207

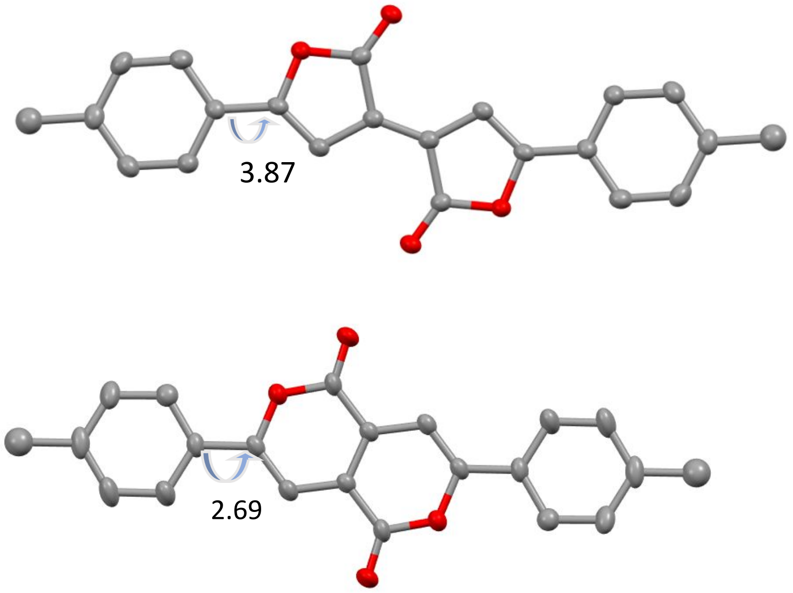

Figure S13. Molecular structure of **O5PMe** and **O6PMe** with isotropic displacement spheres drawn at 50% probability. H atoms are omitted.

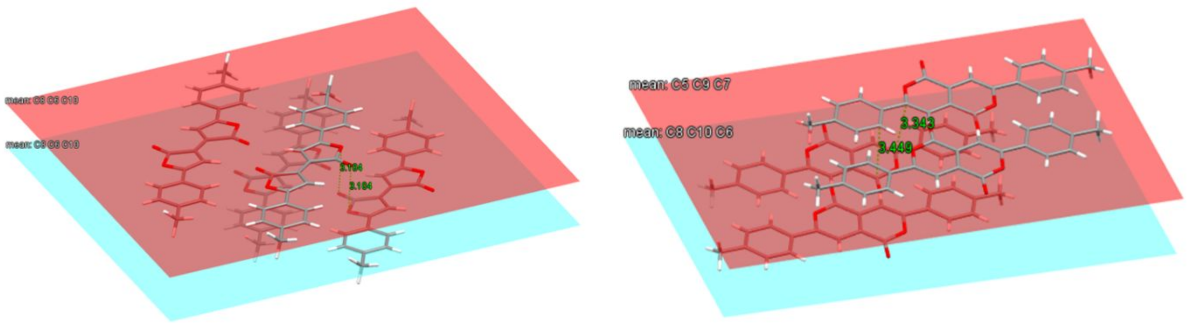

Figure S14. **O5PMe** and **O6PMe** packing arrangement with π–π distance of 3.1 – 3.5 Å between the closest planes of the molecules.

Thin-film Preparation

Thin films of Pechmann dyes were prepared by thermal evaporation in an ultrahigh vacuum environment of 10^{-8} mbar at an evaporation rate of 0.02 nm/s. The rate was monitored using a calibrated quartz crystal microbalance and the deposition was stopped once the desired film thickness (60/100/200 nm) was obtained. Once the evaporation point is attained the vaporized molecules travel from the source to the deposition target where they nucleate and form a thin film coating. High vacuum is used to ensure longer mean free paths to avoid collision with other gas molecules that might be present inside the deposition chamber. The deposition was done onto 1 mm-thick quartz substrates, 0.2 mm-thick glass coverslips and silicon wafers that were cleaned by sequential sonication in acetone and isopropyl alcohol.

Grazing Incidence Wide Angle X-ray Scattering

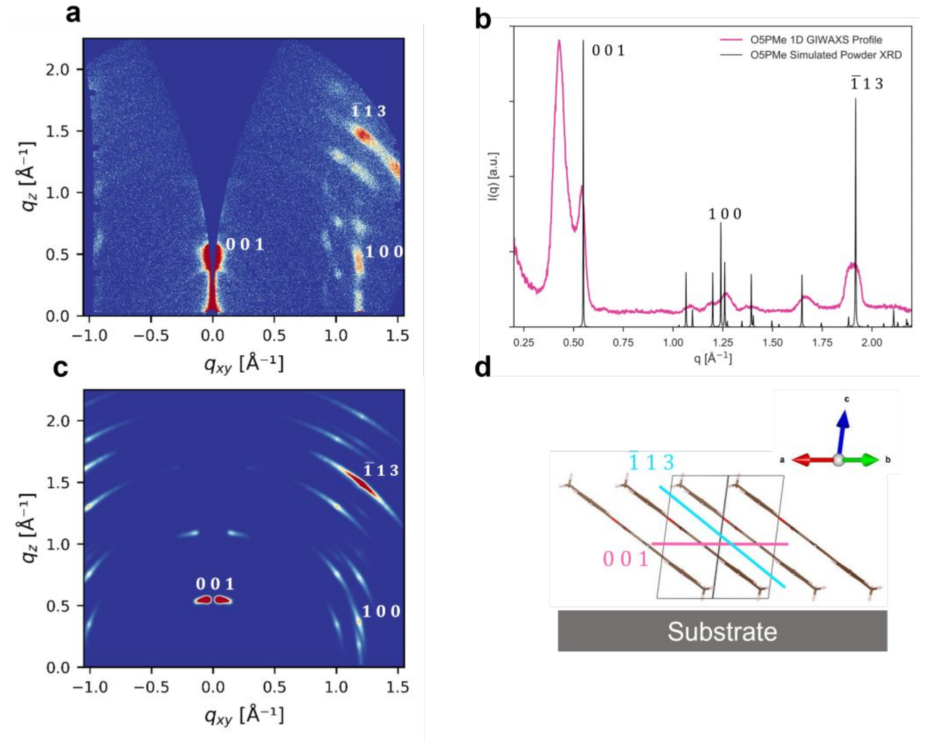

Figure S15: **(a)** 2D GIWAXS pattern of an O5PMe thin film (repeated from Figure 3c). **(b)** Corresponding 1D GIWAXS intensity profile compared with a simulated PXRD profile of the single crystal structure. **(c)** Replicated 2D GIWAXS pattern by simulating a narrow distribution of crystallites with the reciprocal 001 axis oriented along q_z. **(d)** Preferential orientation of O5PMe with the (001) lattice plane aligned parallel to the substrate.

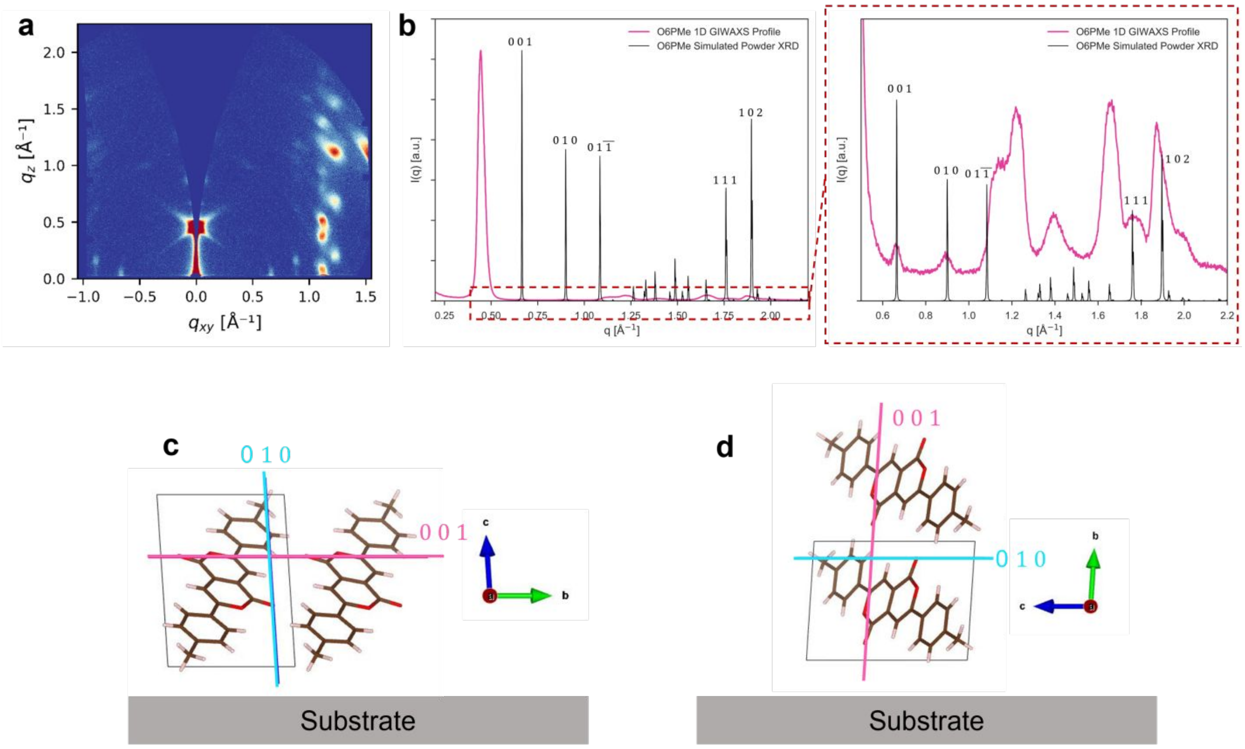

Figure S16: (**a**) 2D GIWAXS pattern of an O6PMe film (repeated from Figure 3d). **(b)** Corresponding 1D GIWAXS intensity profile compared with a simulated PXRD profile of the single crystal structure. The highlighted region at high q indicated by the dashed red rectangle is rescaled in a separate plot for easier viewing. Illustrations of the O6PMe unit cell with the **(c)** (001) and **(d)** (010) lattice plane aligned parallel to the substrate contributing to out-of-plane reflections at q = 0.66 $Å^{-1}$ and q = 0.89 $Å^{-1}$ respectively.

Grazing incidence wide-angle X-ray scattering (GIWAXS) measurements were performed on a Xeuss 2.0 SAXS/WAXS laboratory instrument (Xenocs) equipped with a liquid gallium MetalJet source (Excillum). The source produces a monochromatic X-ray beam with an energy of 9.24keV (λ = 1.34Å) which is directed at sample surfaces in a grazing incidence configuration (incident angle of 0.15°). The scattered X-rays were detected by a vertically offset Pilatus3R 1M (Dectris) 2D X-ray detector positioned ~313 mm from the sample. The sample-to-detector distance was calibrated using a silver behenate standard in transmission geometry. During measurement, the entire flight path including the collimation tubes and sample chamber were held under vacuum to minimize background air scatter. GIWAXS data were corrected, reduced and reshaped using scripts based on pyFAI and pygix python libraries[10]. 1D integrations were performed across the full range of the azimuthal angle normal to the incident beam on the detector ($0° < \chi < 90°$). 2D GIWAXS simulations were performed using the WAXS package in the SimDiffraction MATLAB toolbox[11]. All 2D simulations used a uniaxial model with a Pseudo-Voigt distribution in the out-of-plane direction with the following SimDiffraction parameters: χ angles to probe (-15°:1°:15°), width of out-of-plane distribution $W\perp$ (15°) and lineshape of out-of-plane distribution $\mu\perp$ (0.5). PXRD simulations and crystallographic diagrams were produced in VESTA software using the single crystal structure.

The details of molecular packing, orientation, and crystallinity of films are discussed in the main text and in Figure 3c and 3d where 2D GIWAXS patterns where high intensity Bragg spots can be seen. The features observed for O5PMe match well with the highest intensity features expected for the single crystal structure (e.g. 001, 100 and $\bar{1}13$). The 001 reflection at q = 0.54Å^{-1} is only visible out-of-plane in the 2D GIWAXS pattern indicating that O5PMe preferentially orients with the (001) lattice plane aligned parallel to the substrate as illustrated in Figure S13d. To confirm this, the O5PMe 2D GIWAXS pattern is replicated by simulating a narrow distribution of crystallites with the reciprocal 001 axis oriented along q_z.

The features observed for O6PMe generally do not match well with the features expected for the single crystal structure however we find agreement with some reflections such as those expected for the (001), (010), (111) and (102) lattice plane. Given the (001) and (010) lattice planes are almost orthogonal to each other (α = 91.2690°), the appearance of 001 and 010 reflections in the out-of-plane direction in the 2D GIWAXS pattern at q = 0.66 Å^{-1} and q = 0.89 Å^{-1} respectively suggests that multiple preferential orientations are present in the O6PMe film.

Steady-state Characteristics

Steady-state absorption measurements were performed with a Shimadzu UV3600Plus spectrometer. Steady-state photoluminescence measurements were performed using a custom-built setup with a continuous excitation source. The emission is focused into an Andor Kymera 328i spectrometer and collected using an Andor iDus DU420A BVF Si detector.

Solution state spectra of O6PMe is comparable to its solid-state spectra (Figure S15b), whereas the O5PMe solid-state spectra is hypsochromically shifted with respect to its solution state spectra (Figure S15a).

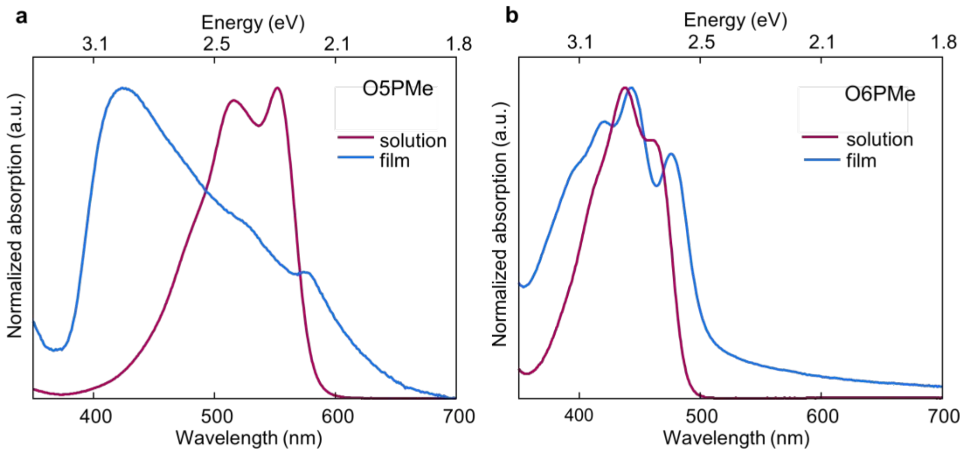

Figure S17: Absorption spectra of a) O5PMe, (b) O6PMe in solution (red) and films (blue).

Time-resolved Emission

To gain insights into the time-resolved dynamics of Pechmann dye films, time-correlated single photon counting measurements were performed. From the two-dimensional map of the time-resolved emission scan of O5PMe ($\lambda_{\_exc}$ = 405nm), two distinct emission components, prompt and delayed are present at early times and the high energy component decays and disappears by 5 ns. The subsequent spectra resemble the steady-state photoluminescence. Furthermore, a redshift in the emission maxima is observed over time. This shift is suggestive of the emission from an intermediate correlated TT pair as also previously been observed for singlet fission systems such as TIPS-tetracene. The decay constants at 520 nm and 650 nm are 3 ns and 13 ns respectively. For O6PMe time-resolved emission ($\lambda_{\_exc}$ = 400nm) however, monoexponential decay is observed over the whole spectral range as shown in Figure S16. The kinetic decay at two emission wavelengths picked for reference are shown in Figure S17b.

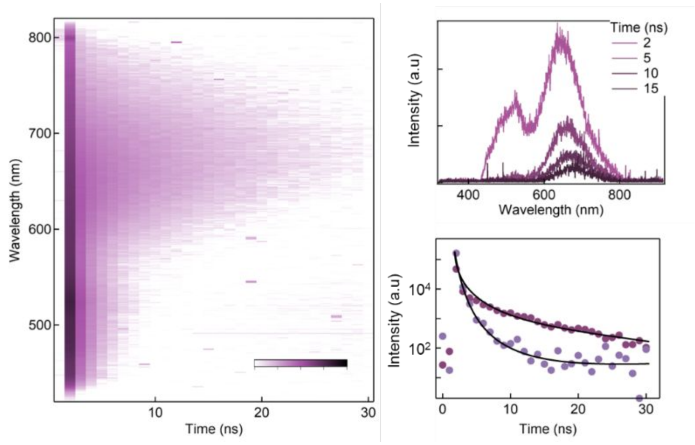

Figure S18: (a) Time-resolved and spectrally resolved emission scan of O5PMe film. **(b)** Spectra at four time-points in the measurement. **(c)** Decay kinetics at 525nm and 650nm.

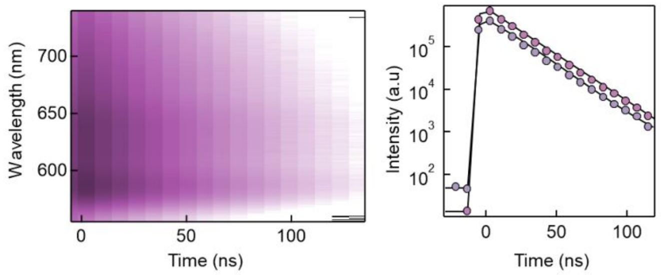

Figure S19: (a) Time-resolved and spectrally resolved emission scan of O6PMe when excited at 400nm. **(b)** Kinetics of decays at 620nm and 720nm shown for reference exhibits monoexponential decay.

Transient Absorption Spectroscopy

Ultrafast transient absorption (TA) measurements were performed using previously reported methods. Depending on the probed spectral range and timescales, different combinations of optical systems were used. Picosecond-scale measurements were performed using an Yb-based 1025 nm laser (PHAROS, Light Conversion) having 400 uJ per pulse with a repetition rate of 38 kHz. The laser output is split into two and one part is modified to create a broadband probe beam by a non-linear white light generation process using a YAG crystal. The second part is used to generate the pump using an optical parametric oscillator system (ORPHEUS-LYRA, Light Conversion). The probe beam is delayed using a computer-controlled mechanical translational stage (Newport). Pump pulses are turned on and off with a mechanical chopper. Both the pulses overlap non-collinearly in the sample and thereafter the transmitted pump beam is blocked to direct only the probe beam to the spectrometer.

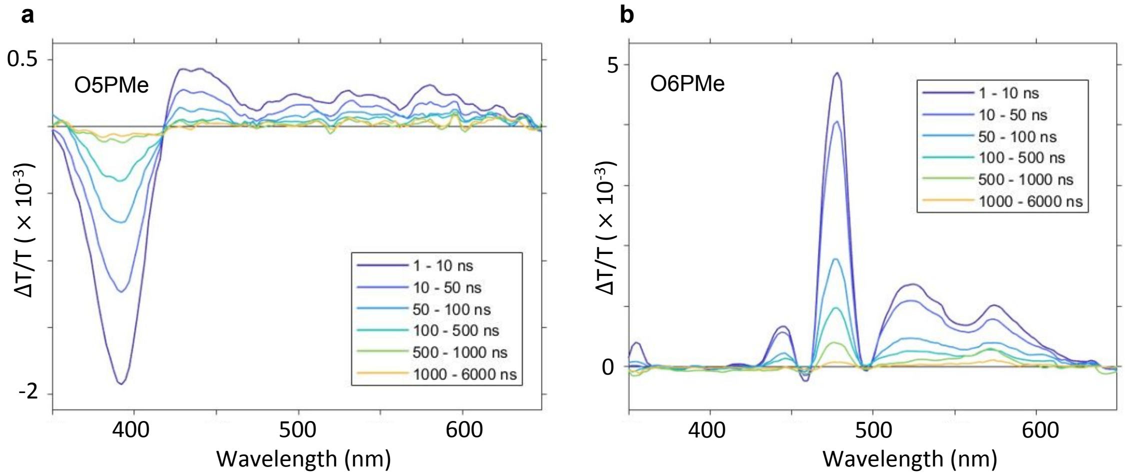

Figure S20: Long-time transient absorption spectra of (**a**) O5PMe and (**b**) O6PMe thin films acquired up to 6μs in a probe spectral region 350 - 660 nm when excited with a short 355nm pump pulse.

Long-time measurements were conducted on a setup pumped by a regenerative Ti:sapphire amplifier (Solstice Ace, Spectra-Physics) centered at 800 nm at a rate of 1 kHz with a total output of 7 W. To perform sub-ns measurements in the ultraviolet range, the output of the amplifier was used to seed a homebuilt broadband NOPA tuned to output 350 - 650 nm pulses generated by focusing the 800 nm fundamental beam onto a CaF_2 crystal (Eksma Optics, 5 mm) connected to a digital motion controller (Mercury C-863 DC Motor Controller) after passing through a mechanical delay stage. The transmitted pulses were collected with a single line scan camera (JAI SW-4000M-PMCL) after passing through a spectrograph (Andor Shamrock SR-163). Nanosecond-scale measurements in the visible range were performed using an 800 nm laser with 0.43 mJ pulse energy and 1 kHz repetition rate. Frequency doubled output of the amplifier was used to seed a homebuilt broadband non-collinear optical parametric amplifier (NOPA) tuned to output 530 - 750 nm pulses with a beta barium borate (BBO) mixing crystal (Eksma Optics). The probe beam is split by a 50% reflectance beam splitter to create a reference. The pump and probe beams are spatially overlapped on the sample adjacent to the

reference beam which is used to account for shot-to-shot variations in probe transmission. The probe and reference beams are then focused into the Shamrock SR-303i, Andor Technology imaging spectrometer and detected with charge-coupled device (CCD) detector arrays (Entwicklungsbüro Stresing).

The short-time transient absorption spectra of both the dyes are shown in the main text Figure 4. The long-time spectra acquired from 1ns to 6μs in the spectral range of 350nm to 660nm follow similar spectral evolution as can be seen in Figure S17. Spectral deconvolution of O5PMe results in two different photoexcited species as shown in Figure S18. The excitation density dependence on the spectral kinetic profiles is investigated by varying the pump excitation power in the range between 50 μW and 550 μW for a laser spot size of 245 μm corresponding to fluences between 5.6 μJcm^{-2} and 61.4 μJcm^{-2}. As shown in Figure S18, the decay kinetics is fluence independent, ruling out thermal artifacts and annihilation processes[12].

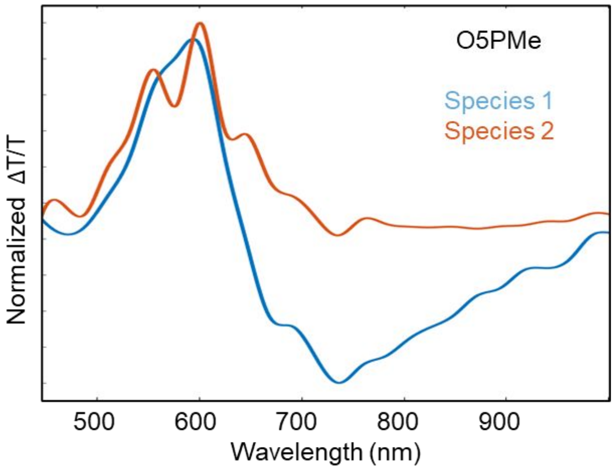

Figure S21: Spectral deconvolution of O5PMe transient absorption spectra presented in Figure 5a in the main text, with two photoexcited species retrieved.

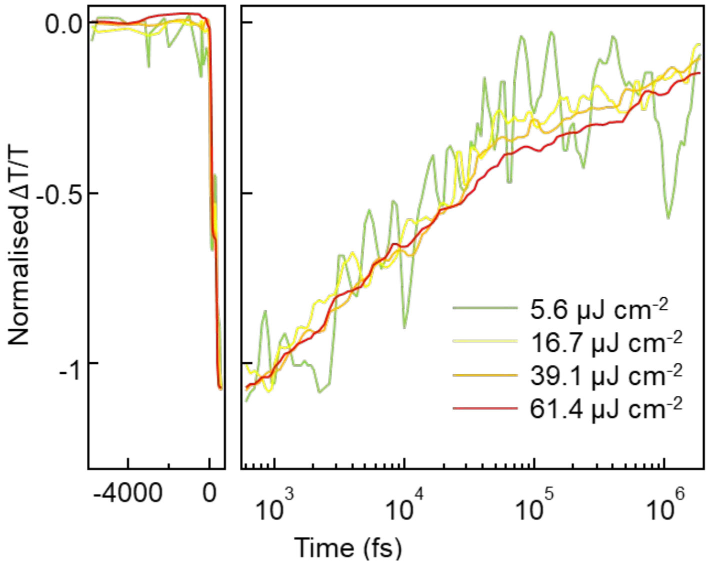

Figure S22: Excitation density dependence of singlet state photoinduced absorption spectral kinetics in O5PMe films. The kinetic profiles at different excitation densities in the range of 5.6 μJcm^{-2} to 61.4 μJcm^{-2} showing no fluence dependence.

Transient Electron Spin Resonance

Time-resolved electron spin resonance (tr-ESR) experiments were conducted using a Bruker Biospin E680 or E580 EleXSys spectrometer operating at X-band (~9.71 GHz), employed with a Bruker ER4118-MD5-W1 dielectric resonator. Signal averaging times varied from eight to more than 16 hours. Temperature was maintained with an ITC-503S temperature controller and a CF-935SW helium flow cryostat (Oxford Instruments). Temperature control was achieved through liquid nitrogen or helium flow, with control facilitated by the Oxford Instruments ITC-503S temperature controller. Sample light excitation was provided with an Opolette HE355 OPO operating at 20 Hz, with a pulse energy of 1-2 mJ. Tr-ESR spectra were simulated using the EasySpin toolbox in MATLAB™ to extract ZFS parameters and sublevel populations[13].

References

[1] M. Hayashi, F. Toshimitsu, R. Sakamoto, H. Nishihara, *J. Am. Chem. Soc.* **2011**, *133*, 14518.

[2] M. M. Martínez, C. Pérez-Caaveiro, M. Peña-López, L. A. Sarandeses, J. P. Sestelo, *Org. Biomol. Chem.* **2012**, *10*, 9045.

[3] A. Stanger, *J. Org. Chem.* **2006**, *71*, 883.

[4] D. Geuenich, K. Hess, F. Köhler, R. Herges, *Chem. Rev.* **2005**, *105*, 3758.

[5] E. Matito, *Univ. Girona Spain Euskal Herriko Unibersitatea Spain*.

[10] G. Ashiotis, A. Deschildre, Z. Nawaz, J. P. Wright, D. Karkoulis, F. E. Picca, J. Kieffer, *J. Appl. Crystallogr.* **2015**, *48*, 510.

[11] D. W. Breiby, O. Bunk, J. W. Andreasen, H. T. Lemke, M. M. Nielsen, *J. Appl. Crystallogr.* **2008**, *41*, 262.

[12] K. J. Fallon, P. Budden, E. Salvadori, A. M. Ganose, C. N. Savory, L. Eyre, S. Dowland, Q. Ai, S. Goodlett, C. Risko, D. O. Scanlon, C. W. M. Kay, A. Rao, R. H. Friend, A. J. Musser, H. Bronstein, *J. Am. Chem. Soc.* **2019**, *141*, 13867.

[13] Stoll, S.; Schweiger, A. EasySpin, a comprehensive software package for spectral simulation and analysis in EPR. *J. Magn. Reson.* **2006**, 178, 42–55.
